# Diversity and metabolism of *Woeseiales* bacteria, global members of marine sediment communities

**DOI:** 10.1038/s41396-020-0588-4

**Published:** 2020-01-27

**Authors:** Katy Hoffmann, Christina Bienhold, Pier Luigi Buttigieg, Katrin Knittel, Rafael Laso-Pérez, Josephine Z. Rapp, Antje Boetius, Pierre Offre

**Affiliations:** 1 HGF-MPG Joint Research Group for Deep Sea Ecology and Technology, Max Planck Institute for Marine Microbiology, Bremen, Germany; 2 HGF-MPG Joint Research Group for Deep Sea Ecology and Technology, Alfred-Wegener-Institut Helmholtz-Zentrum für Polar- und Meeresforschung, Bremerhaven, Germany; 3 Department of Molecular Ecology, Max Planck Institute for Marine Microbiology, Bremen, Germany; 4 MARUM - Center for Marine Environmental Sciences, University of Bremen, Bremen, Germany; 5 NIOZ Royal Netherlands Institute for Sea Research, Department of Marine Microbiology and Biogeochemistry, and Utrecht University, Den Burg, The Netherlands; 6 Present Address: School of Oceanography, University of Washington, Seattle, WA 98185 USA

**Keywords:** Microbial ecology, Marine microbiology

## Abstract

Surveys of 16S rRNA gene sequences derived from marine sediments have indicated that a widely distributed group of *Gammaproteobacteria*, named “JTB255-Marine Benthic Group” (now the candidate order *Woeseiales*), accounts for 1–22% of the retrieved sequences. Despite their ubiquity in seafloor communities, little is known about their distribution and specific ecological niches in the deep sea, which constitutes the largest biome globally. Here, we characterized the phylogeny, environmental distribution patterns, abundance, and metabolic potential of *Woeseiales* bacteria with a focus on representatives from the deep sea. From a phylogenetic analysis of publicly available 16S rRNA gene sequences (≥1400 bp, *n* = 994), we identified lineages of *Woeseiales* with greater prevalence in the deep sea than in coastal environments, a pattern corroborated by the distribution of 16S oligotypes recovered from 28 globally distributed sediment samples. Cell counts revealed that *Woeseiales* bacteria accounted for 5 ± 2% of all microbial cells in deep-sea surface sediments at 23 globally distributed sites. Comparative analyses of a genome, metagenome bins, and single-cell genomes suggested that members of the corresponding clades are likely to grow on proteinaceous matter, potentially derived from detrital cell membranes, cell walls, and other organic remnants in marine sediments.

## Introduction

Marine sediments cover ~70% of Earth’s surface, hosting a huge diversity of bacterial populations that gain energy and nutrients from the remineralization of sedimented organic matter [1, 2]. The myriad of remineralization processes taking place in marine sediments play major roles in the global carbon cycle by influencing the sequestration of carbon in the seafloor and the recycling of nutrients to fuel primary production in the sea. Although the kinetics of labile components of sedimentary organic matter (e.g., carbohydrates, proteins, and lipids) has been investigated to some extent, there are considerable gaps in our knowledge of the key microbial agents mediating these processes [2]. The identity and relative contributions of the major groups of microorganisms involved in the degradation of sedimentary biomacromolecules remain unknown. This is further emphasized by the high degree of spatial heterogeneity of the seafloor in microbial community composition and organic matter deposition, recycling, and burial in different regions of the seafloor, e.g., between shallow coastal sediments and deep oceanic sediments [2, 3].

Recent surveys of 16S rRNA gene sequences have revealed that the largely uncultivated group of *Gammaproteobacteria* formerly known as “JTB255-Marine Benthic Group” (abbreviated JTB255-MBG) is globally distributed in both coastal and deep-sea sediments [4–7]. In these studies, members of this group account for 1–22% of the sequences retrieved. Cell counts in coastal sediments have indicated that these bacteria account on average for 6% of the surveyed microbial communities [6, 8, 9], yet their cell densities in deep-sea sediments remained unknown. Notably, distinct lineages within JTB255-MBG were detected in coastal and deep-sea sediments, suggesting that certain taxa within this group occur in specific types of environments, i.e., in either deep-sea or coastal environments [8]. However, this hypothesis has been based on a limited number of observations. Here we use the taxon name *Woeseiales* (a candidate order) to collectively refer to the group that includes JTB255-MBG and the strain *Woeseia oceani* XK5 [10], as proposed in the Genome Taxonomy Database (GTDB) [11], which provides a standardized taxonomy based on genome sequences. The initial placement of *W. oceani* XK5 within *Chromatiales* [10] is not supported in both GTDB and the SILVA database [11, 12], and *Woeseiales* (*sensu* GTDB) appears to be synonymous with *Woeseiaceae* [8, 10] (*sensu* SILVA). The exact placement of *Woeseiales*/*Woeseiaceae* within *Gammaproteobacteria* remains, however, uncertain. Although previous studies have noted the organoheterotrophic metabolism of *W. oceani* XK5 [8, 10], investigations targeting uncultivated members of *Woeseiales* in coastal sediments have emphasized their potential for facultative chemolithoautotrophy, possibly driven by the oxidation of hydrogen and inorganic sulfur compounds [8, 13]. In this study, we focus on the members of *Woeseiales* inhabiting deep-sea surface sediments, and assess the phylogeny, environmental distribution, abundance, and metabolic potential of these microorganisms. The main hypotheses tested are (I) different lineages within *Woeseiales* are associated with deep-sea or coastal environments, (II) they are a major component of the microbiome of deep-sea surface sediments, and (III) the genomic traits of the members of deep-sea lineage(s) indicate a chemoorganoheterotrophic lifestyle targeting complex detrital biomolecules.

## Materials and methods

### Samples

**Fig. 1:**
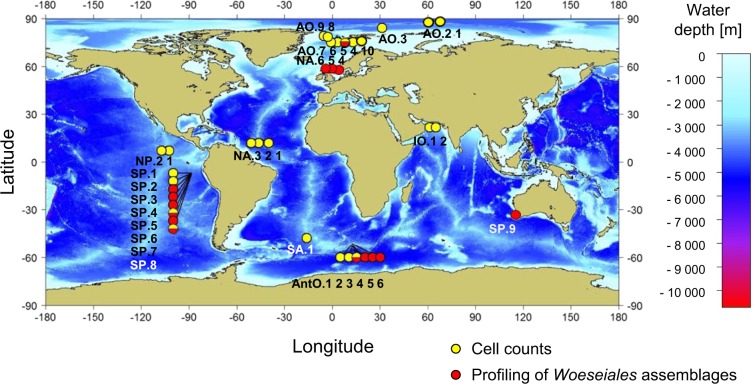
Geographic location of sampling sites investigated in this study. Sampling sites are indicated on the map using circular markers. Site identifiers are indicated next to the corresponding markers, and characteristics of the respective sites are listed in Table S1. The type of analysis performed on the samples recovered from the different sites was directly indicated by color coding of the location markers: cell counts including total cell counts as well as counts of gammaproteobacteria and *Woeseiales* bacteria (yellow); profiling of the composition of *Woeseiales* assemblages (red). The use of a sample recovered from the AO.5 site for metagenomic and single-cell genomic analyses of *Woeseiales* bacteria was not indicated on the map. The depth of the ocean seafloor was color coded according to the color scale on the right-hand side of the map: the scale unit is in meters below the ocean surface. The map was generated with the R package “marmap” [70].

Multicorers, ROV-operated push corers, and gravity corers were deployed to sample sediments from the undisturbed seafloor of several oceanic regions. These included the Arctic, South and North Pacific, South and North Atlantic, Indian, and Antarctic Ocean, at water depths between 75 and 5500 m (Table S1). We acquired additional samples from the water column by using conductivity, temperature, and depth rosette instruments equipped with 12 L Niskin bottles (Table S1). Geographic location of samples investigated in this study is shown in Fig. 1. Information about all newly collected samples and the corresponding metadata was submitted to the World Data Center PANGAEA (https://doi.pangaea.de/10.1594/PANGAEA.884844).

### Microbial cell counts

We fixed 0.5 g aliquots of sediment with a 4% formaldehyde solution for 2–4 h, and washed the fixed sediments three times with 1× phosphate-buffered saline (PBS), before storing them in 50% ethanol/PBS at −20 °C. For water samples, we fixed 10 ml (for samples from the deep chlorophyll maximum and 100 m water depth) and 30 ml (for meso- and bathypelagic samples) samples with formaldehyde to a final concentration of 2–4% for 2–4 h, then filtered the fixed water samples over 0.22 µm polycarbonate filters, and stored the filters at −20 °C until further use. We performed total cell counts as described by Schauer et al. [14] using the nucleic acid dye 4′-6-diamidino-2-phenylindole (DAPI) and an Axio Imager M1 epifluorescence microscope (Zeiss, Oberkochen, Germany) equipped with a 100 × /1.25 oil plan-apochromat objective. We then performed catalyzed reporter deposition–fluorescence in situ hybridization (CARD–FISH) according to Ishii et al. [15] to visualize cells of *Woeseiales* bacteria and *Gammaproteobacteria* on the filters we used for total cell counts. We used a mix of novel CARD–FISH probes, including two horseradish peroxidase-labeled probes referred to as JTB819 and JTB897, and a competitor oligonucleotide referred to as cJTB897 (Supplementary text 1 and Table S2). We used the GAM42a oligonucleotide probe and the cBET42a competitor oligonucleotide to visualize the members of *Gammaproteobacteria* [16]. Previous tests showed that the GAM42a probe does not target several gammaproteobacterial groups [17] including *Woeseiales* [6]. Hence, total counts of gammaproteobacteria were estimated as the sum of all cells detected with the GAM42a probe and our new probe mix, using duplicate filters for each of the investigated samples (see Table S3). A list of all processed samples and numbers of replicate filters can be found in Table S3. Cells were generally counted in 20 independent grids, except in cases where a total of 1000 DAPI-stained cells were counted in fewer than 20 grids, in which case cells were counted in the number of grids sufficient to count 1000 DAPI-stained cells. Median cell counts per filter were 1026 (range: 251–1514) DAPI-stained cells, 47 (range: 3–135) JTB819/JTB897-positive cells, and 38 (range: 2–195) Gam42a-positive cells. We estimated the global abundance of *Woeseiales* bacteria and gammaproteobacteria by extrapolating the average cell densities in deep-sea surface sediments (upper 10 cm) that we determined from samples collected at 23 globally distributed sites (Fig. 1, Tables S1, S2) to the global volume of deep seafloor sediment (upper 10 cm) below 200 m of water depth (Supplementary text 2). We used linear regression, supported by Pearson and Spearman correlation statistics, to explore the relationships between the determined densities of *Woeseiales* bacteria in the surveyed sediment samples, and both water depth and latitude of the sampling sites (Table S1). A similar approach was used to investigate the relation between the densities of *Woeseiales* bacteria in the sediment samples and total organic carbon (TOC) and primary productivity data reported in ref. [18]. Furthermore, a general linear mixed model created with the R package lmerTest [19, 20] was used to evaluate the relationship between the densities of *Woeseiales* bacteria and sediment depth and oxygen concentration (i.e., fixed-effect factors) derived from eight and four sediment cores, respectively (i.e., the random effect factor).

### Environmental distribution of 16S rRNA gene sequences of *Woeseiales* bacteria

We investigated the environmental distribution of 16S rRNA gene sequences of *Woeseiales* bacteria in order to evaluate whether distinct taxa within *Woeseiales* associate with different environments, with a main focus on deep-sea and coastal environments. Our investigation was based on two independent but complementary approaches: (i) a survey of the environmental origin of all near-full-length 16S rRNA gene clone sequences (≥1400 bp; *n* = 997) assigned to JTB255-MBG in SILVA release 128 [12], and (ii) a comparative analysis of the oligotype profiles [21, 22] derived from the high-throughput sequencing of 16S rRNA gene amplicons obtained from 28 samples, including 21 samples from deep-sea sites and 7 from coastal sites (Table S1). While the first approach covers a large number of sites at the cost of a shallow sampling of the diversity of *Woeseiales* bacteria at the sampled sites, the second approach focuses on fewer sites but with the advantage of a much deeper coverage of the diversity of *Woeseiales* bacteria. For both approaches, the following categories were used to classify the sampling sites: (1) deep-sea environments (i.e., any marine environment located at least 200 m below the sea surface, including both benthic and pelagic environments), (2) environments of the marine littoral, hereafter referred to as coastal environments (any marine environment located no further than 200 m below the sea surface, and including benthic, pelagic, and tidal environments), and (3) continental environments including soil, river, and lake samples (used solely for the first approach).

#### Approach 1

We grouped the 16S rRNA gene clone sequences we collected from SILVA into two types of taxonomic units in order to evaluate taxon-environment associations within *Woeseiales* at different taxonomic scales. We first grouped the sequences into phylotypes (i.e., finer taxonomic units) using a hierarchical clustering approach and then grouped the phylotypes into lineages (i.e., coarser taxonomic units comprising a minimum of two phylotypes) using a maximum likelihood phylogenetic analysis. To generate the phylotypes, we first downloaded from the SILVA SSUref database an alignment of the near full length 16S rRNA gene clone sequences assigned to JTB255-MBG and filtered out sequences with homopolymeric regions of eight or more nucleotides, resulting in a set of 994 sequences. Uncorrected pairwise distances were then calculated between the 994 clone sequences remaining in the alignment, and by applying a hierarchical furthest-neighbor clustering [23] at 96% sequence identity using mothur v1.37.6 [24], we produced a total of 171 phylotypes (Table S4). One sequence per phylotype was then picked (i.e., the first accession number in each phylotype) for subsequent phylogenetic analyses (Supplementary text 3) as it allows filtering out highly similar sequences that generally lead to short branches manifesting weak phylogenetic signal.

We then examined the environmental origin of the sequences within each phylotype and lineage to evaluate whether these taxa are overrepresented in any of the three environmental categories we used to classify sequence-sampling sites. Descriptions of the environmental origin of the clone sequences were retrieved from SILVA by parsing the attributes “isolation source” and “reference title” in each sequence entries. When attribute values (Table S5) were not clear enough to classify the sampling sites into the deep-sea, coastal, or continental environment categories, we consulted the publications linked to the corresponding sequence entry in SILVA. We were able to classify the sampling sites of 933 clone sequences (of a total of 994) into one of the three environmental categories we used in our classification scheme. We investigated associations between phylotypes and environmental categories further by performing a correspondence analysis (CA) of an environment × phylotype matrix (see Supplementary text 4 for the methods and Table S6 for the data matrix).

#### Approach 2

We compared the composition of assemblages of *Woeseiales* bacteria in samples collected from deep-sea and coastal environments to determine whether some of the *Woeseiales* taxa in the assemblages we surveyed were present in only one type of samples (i.e., either deep-sea or coastal samples). Deep-sea sampling sites were located in the Arctic, Antarctic, and South Pacific Oceans and represented four subcategories of deep-sea environments including deep-sea surface sediments (0–2 cm below seafloor surface), deep-sea subsurface sediments (>2 cm below seafloor surface), deep-sea polymetallic nodules, and deep-sea waters. Coastal sites were located in the North Atlantic and South Pacific Oceans and represented a single subcategory of coastal environments; i.e., coastal surface sediments (0–2 cm below seafloor surface). We grouped the 16S amplicon sequences into oligotypes, which offer a higher degree of taxonomic resolution than the phylotypes and lineages we used for the analysis of 16S clone sequences (methods for the grouping of 16S amplicon sequences into oligotypes and comparison of oligotype profiles are described in detail in Supplementary text 5 and Supplementary files 1–4). We then tested whether the patterns of environmental distribution we observed for the *Woeseiales* oligotypes support those we inferred for the *Woeseiales* phylotypes and lineages (see Approach 1 above). For that purpose, we evaluated the evolutionary placement of the *Woeseiales* oligotypes using the evolutionary placement algorithm [25] implemented in RAxML v8.2.X [26] and based on this analysis assigned the oligotypes to the phylotypes and lineages we defined based on near full-length 16S rRNA gene sequences (see Approach 1 above). Methods for the evolutionary placement and assignment of the *Woeseiales* oligotypes are further described in Supplementary text 6 (see also Supplementary file 5 for the raw data file).

### Single-cell genomics

Arctic deep-sea surface sediments were collected at the LTER HAUSGARTEN in Fram Strait in June 2014 (AO.5a; Fig. 1 and Table S1). Upon retrieval, we diluted sediments with 0.22 µm-filtered seawater in a 1:1 (v:v) ratio, stored them at 4 °C and later shipped aliquots of 20 mL at 5 °C to the Bigelow Laboratory Single Cell Genomics Center (SCGC). At the SCGC, cells were sorted in a total of eight 384-well plates and lysed at 4 °C (four plates) and 20 °C (four plates) using KOH [27]. Detailed procedures for single-cell sorting, cell lysis, multiple displacement amplification, cell screening, genome sequencing, and assembly are provided in Swan et al. [28], on the SCGC’s website (https://scgc.bigelow.org), and in Supplementary text 7 (see also Supplementary file 6). We retained two single-cell genomes referenced as AG-115_M06 + AF-234_H05 and AG-113_B02 + AF-233_C01 (subsequently referred to as M06 and B02, respectively) for further study.

### Metagenome assembly and binning

We assembled and binned genome fragments of *Woeseiales* bacteria from a shotgun metagenome derived from Arctic deep-sea surface sediments collected at the LTER HAUSGARTEN (AO.5a; Fig. 1 and Table S1). We extracted DNA from three 0.5 g sediment aliquots prepared from the top centimeter of one sediment core using a MoBio PowerSoil Kit (MoBio Laboratories Inc., Carlsbad, CA, USA). DNA extracts were treated with RNase (Fermentas, now ThermoFisher Scientific, Waltham, MA, USA), further purified with the Genomic Clean & Concentrator Kit (Zymo Research, Irvine, CA, USA), and subsequently pooled (1.0 µg DNA in total) for the preparation of an Illumina TruSeq sequencing library (insert size: 400 bp, 2 × 300 bp paired-end reads). The library was sequenced at CeBiTec (University of Bielefeld) on an Illumina MiSeq platform generating 4,108,971 paired-end reads. Trimming, quality filtering, and merging of the reads were performed with BBTools v. 35.68 (Bushnell B.— sourceforge.net/projects/bbmap/), and the processed reads were assembled using SPAdes v3.6 [29] as specified in Supplementary file 7.

We used Metawatt [30] to bin and taxonomically classify assembled genome fragments using default parameters, except for the binning step that was performed using the high-confidence level setting. The binning procedure was based on the GC-content, tetranucleotide frequencies, and sequencing depth of the genome fragments, while the taxonomic classification of the resulting bins was performed with a BLASTP-dependent classifier. Prior to the taxonomic classification of the bins, we extended the reference genome database of Metawatt with publicly available genomes (both partial and complete) of *Woeseiales* bacteria: the closed genome of *W. oceani* XK5 (NCBI Reference Sequence NZ_CP016268.1), a partial single cell genome (IMG Genome ID 2651869504), our partial, M06, single cell genome (see above), and two metagenome bins (IMG Genome ID 2651869885 and 2695420981) [8]. Two of the bins we recovered were assigned to *Woeseiales*. Those bins were named bin1_HGIV and bin2_HGIV. We performed targeted reassembly of our candidate *Woeseiales* bins using BBMap v. 35.68 for mapping reads of the unassembled metagenomes onto the contigs of the draft bins, and SPAdes v3.6 run with the flags–only assembler and–careful for the assembly of the mapped reads, a process repeated four times with increasing read-mapping stringency from 95% sequence identity (first two reassemblies) to 98% (last two reassemblies). Scaffolds with a sequence length <500 bp were excluded. We assessed completeness, redundancy, and contamination of the bins and single cell genomes (see previous paragraph) with CheckM using the gene marker set for *Gammaproteobacteria* [31]. Phylogenetic placement of the recovered genomes was inferred as specified in Supplementary text 8.

### Metabolic reconstruction and comparative genome analyses

The metabolism of *W. oceani* XK5 was reconstructed using a comparative genome annotation approach. The metabolic reconstruction focused on the energy metabolism of *W. oceani* XK5 including possible respiratory chains, catabolic pathways, central metabolic pathways, uptake transporters, hydrolytic enzymes and their secretion systems. Possible components of the investigated pathways and protein complexes were searched among all proteins that were predicted from the genome of *W. oceani* XK5, the sequence of which is publicly available in RefSeq [32, 33]. First, we annotated the predicted proteome of *W. oceani* XK5 with protein domains using the hmmscan algorithm (reporting e-value threshold: ≤1e–5; reporting conditional e-value threshold: ≤1e–5) implemented in HMMER 3.1b2 [34, 35] and the library of hidden Markov models of the Pfam protein family database release 30.0 [36]. Then, *W. oceani*’s proteins matching the Pfam domain annotations of biochemically characterized proteins, referenced in MetaCyc [37], TCDB [38], MEROPS [39], CAZy [40], and ESTHER [41] were further investigated to evaluate their relatedness to well-studied enzymes/proteins and assess their putative function. The functional annotation of candidate proteins was further curated by inspecting (i) the presence of additional/missing protein domains via a comparison of the domains of *W. oceani*’s candidate proteins and reference (i.e., biochemically characterized) proteins using CD search [42] and InterProScan [43], (ii) the assignment of the candidate proteins to a cluster of orthologous groups [44], (iii) pairwise protein–protein sequence alignments with homologous proteins, and (iv) the presence of a signal peptide and membrane-spanning domains using SignalP [45] and TMHMM 2.0c [46], respectively.

The repertoire of putative peptidases encoded in the genome of *W. oceani* XK5 was then compared with the repertoires of putative peptidases encoded in draft genomes currently available for the order *Woeseiales*, in order to evaluate functional variability between distinct members of *Woeseiales*. Proteins encoded in the newly obtained M06 and B02 single-cell genomes and bin1_HGIV and bin2_HGIV metagenome bins were predicted using Prodigal v 2.6.1 [47] with the –p flag selected. Proteins predicted from the previously published SAG 1868_B (IMG Genome ID 2651869504) and metagenome bins bin20_j1 (IMG Genome ID 2651869885) and WOR_SG8_31 (Genbank accession LJTI00000000) were downloaded from the corresponding public databases. Putative peptidases were then searched in the predicted proteomes using the default settings of the phmmer algorithm of HMMER 3.1b2 and the merops_scan sequence library as reference database. Putative proteins matching non-peptidase homologs and peptidase inhibitors referenced in the merops_scan sequence library were not reported.

### Data accessibility

Cell abundance data were submitted to PANGAEA (doi.pangaea.de/10.1594/PANGAEA.874860). Genomic data, including single-cell genomes M06 (GCA_902167425) and B02 (GCA_902167415), and metagenome bins bin1_HGIV and bin2_HGIV, are available under INSDC accession: Bioproject ID PRJEB20570. For the accession numbers of other genomic data, including short paired-end, primer-trimmed reads, see Table S1. The data were archived using the brokerage service of GFBio [48].

## Results and discussion

### Phylogenetic placement of the order *Woeseiales*

**Fig. 2:**
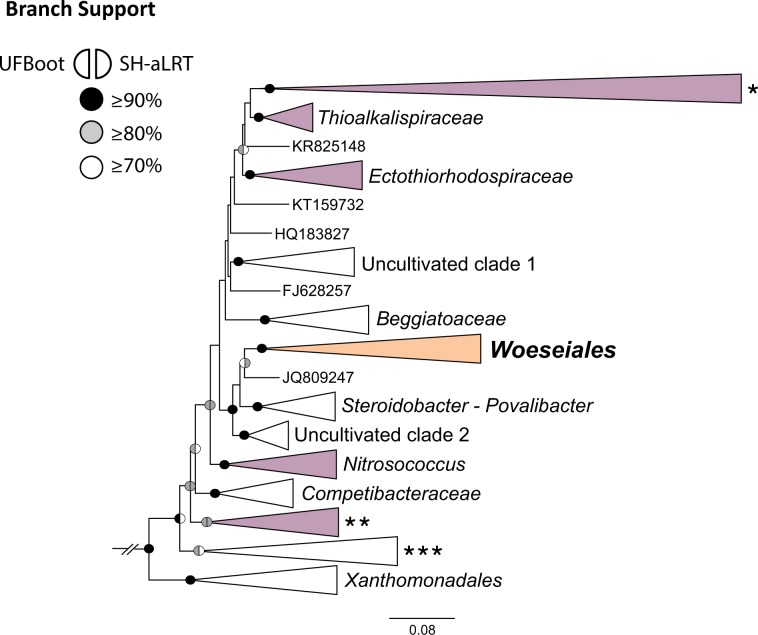
Phylogenetic placement of *Woeseiales*. The phylogeny is based on an alignment of 919 sequences (≥1400 bp) of the 16S rRNA gene. These sequences cover all major, validly described, gammaproteobacterial orders including *Woeseiales* and few betaproteobacteria (outgroup). Sequence accession numbers and their taxonomic classification in the SILVA database release 128 are shown in Table S7. The phylogeny was inferred by maximum likelihood using the GTR model of nucleotide substitution, and ten rate categories unconstrained by a probability distribution (GTR + R10). Scale bar indicates the number of nucleotide substitutions per site. Branch support values are represented as color-coded circles (see symbol key on the figure) and were only indicated for those branches that had a support value ≥70% using both methods. Three unnamed clades (i.e., *, **, and ***) include sequences from multiple orders of *Gammaproteobacteria* as indicated in Table S7. Purple-colored clades include sequences of organisms that are usually accommodated in *Chromatiales*. Clades have been collapsed for better readability.

We investigated the phylogenetic placement of 171 phylotypes, which cover the diversity of all 16S rRNA gene clone sequences (≥1400 bp) assigned to JTB255-MBG in SILVA 128. The phylotypes formed a well-supported clade placed among the basal lineages of *Gammaproteobacteria* (Fig. 2). This clade was part of a larger lineage that included the clades referred to as “*Steroidobacter*–*Povalibacter*” and “Uncultivated clade 2” in Fig. 2. The latter two clades comprise sequences from *Steroidobacter denitrificans* [49], *Povalibacter uvarum* [50], and clones derived from continental environments as indicated in the corresponding sequence entries in SILVA (see Table S7 for a complete listing of accession numbers). The latest releases of SILVA and GTDB [11, 12] also support the clustering of *Woeseiales* with the “*Steroidobacter*–*Povalibacter*” clade: these two clades are sister families in SILVA (i.e., *Woeseiaceae* and *Steroidobacteraceae*) and sister orders in GTDB. We observed that some of the sequences we analyzed shared as little as 84% sequence identity, supporting the ranking of the *Woeseiales* clade as a taxonomic order, if minimum sequence identity reported by Yarza et al. [51] is followed. The tentative placement of the *Woeseiales* clade within *Chromatiales* [10] is, however, not supported by our analysis, SILVA and GTDB, and its position relative to other basal lineages of *Gammaproteobacteria* remains so far unresolved (Supplementary text 9).

### Taxon-environment associations within the order *Woeseiales*

**Fig. 3:**
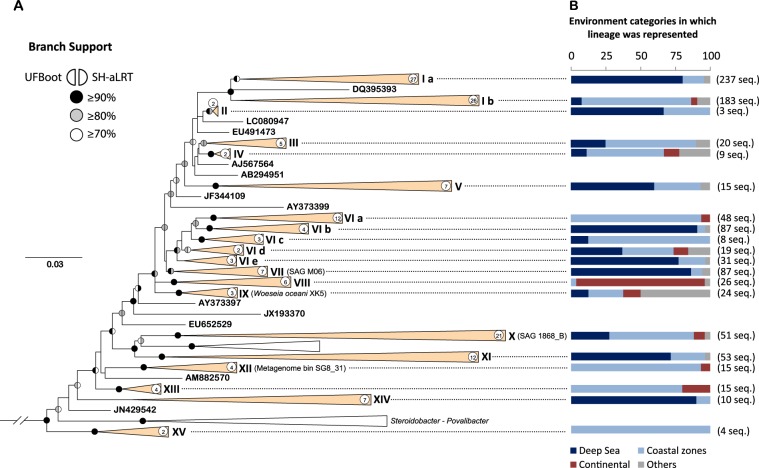
Phylogeny and environmental origin of 16S rRNA gene clone sequences (≥1400 bp) assigned to *Woeseiales*. **a** Phylogenetic tree inferred by maximum likelihood using the GTR model of nucleotide substitution, and seven rate categories unconstrained by a probability distribution (GTR + R7). Scale bar indicates the number of nucleotide substitutions per site. Branch support values were represented as color-coded circles (see symbol key on the figure) and were only indicated for those branches that had a support value ≥70% using both methods. All sequences within collapsed, orange colored, lineages as well as all singletons are assigned to *Woeseiales* whereas sequences within collapsed, white colored, lineages are assigned to *Incertae Sedis* and uncultivated taxa within *Xanthomonadales*, in the SILVA database release 128. Accession numbers of all sequences used in this phylogeny are reported in Table S7. The gammaproteobacterial order *Nevskiales* was used as outgroup (outgroup has been masked). Numbers in white circles indicate the number of phylotypes within the collapsed (orange-colored) lineages and accession numbers of the corresponding sequences (including lineages’ type sequences) are listed in Table S8. Identifiers of genomes investigated in this study (Table S13) are indicated in parenthesis next to lineages VII, IX, X, and XII to indicate the phylogenetic placement of the genomes as determined by their respective 16S rRNA gene sequence. **b** Bar plots representing the environmental origin of 16S rRNA gene sequences (≥1400 bp) assigned to JTB255-MBG in SILVA 128. Each of the bar plots represents the environmental origin of a subset of sequences assigned to a particular lineage of *Woeseiales* (total number of sequences assigned to the lineages are indicated next to the plots). Color motifs of the bar plots indicate the proportion of sequences recovered from four categories of environments (see color key on the figure and Table S8 for detailed sequence counts and number of studies from which the sequences were derived).

We examined the environmental distribution of lineages within the order *Woeseiales*. Our phylogenetic analysis clustered 161 of the phylotypes we retrieved into 15 distinct lineages and placed the ten remaining phylotypes as singleton nodes (Fig. 3a, Table S8). Between 2 and 54 phylotypes were grouped within each of the 15 lineages, the type sequences of which are indicated in Table S8. Two of the lineages (i.e., lineages I and VI) were subdivided into two and five sublineages, respectively. The compilation and categorization of the sampling sites from which the clone sequences were retrieved clearly indicated that members of several lineages were differentially represented across the environment categories (Fig. 3b). For example, most sequences assigned to lineages Ia (190 of 237), VIb (79 of 87), and VII (75 of 87) were obtained from deep-sea environments, most of those assigned to lineages Ib (146 of 183) and VIa (45 of 48) were retrieved from coastal environments, and most sequences assigned to lineage VIII (24 of 26) were obtained from continental environments (including inland waters, sediments, and soils). We observed similar evidence of taxon-environment associations at the finer taxonomic scale of the phylotypes, but did not find strong evidence for strict phylotype-environment associations (Table S6). Environmental distribution of the 171 phylotypes we investigated was visualized using a CA (Figs. S1 and S2), which is further discussed in Supplementary text 10.

**Fig. 4:**
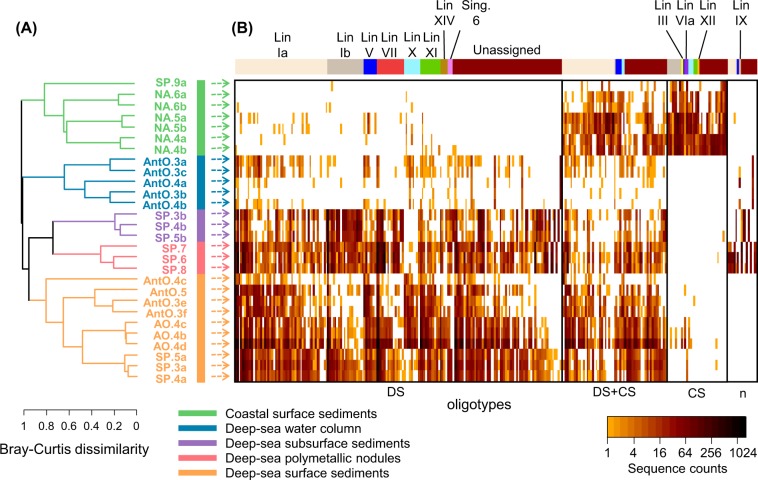
Oligotyping of *Woeseiales* assemblages in marine sediment and water samples. **a** Dendrogram depicting the hierarchical clustering of oligotype profiles obtained from marine sediment and water samples. The dendrogram was generated from the complete-linkage hierarchical clustering of the Bray–Curtis dissimilarity calculated from relative sequence proportions. The environmental origin of the analyzed samples was color coded as directly indicated on the figure by the color key. Geographic location of the analyzed samples is indicated in Fig. 1 by their short identifiers and characteristics of the sampling sites are further described in Table S1. **b** Heat map illustrating sequence counts for 288 oligotypes resolved from 16S rRNA gene sequence tags (V3–V4 region) assigned to *Woeseiales*. Oligotypes were organized according to their distribution patterns in the samples we surveyed and categorized into four groups highlighted with black contour lines: a group of deep-sea oligotypes (DS), a ‘ubiquitous’ group (DS + CS), a group of coastal oligotypes (CS), and a group including all remaining oligotypes (*n*). Within these four groups, oligotypes were sorted according to their placement within the lineages shown in Fig. 3 as indicated by the colored bars above the heatmap (see Table S10 for details). Sequence counts reported on the heatmap for each oligotype are color coded with darker color shades indicating higher sequence counts than lighter color shades. The non-detection of an oligotype was represented as a white space on the heat map.

We used a different approach to examine environmental associations of taxa within *Woeseiales* at a finer taxonomic scale. This was based on the comparison of the oligotype profiles of assemblages of *Woeseiales* bacteria in 21 deep-sea samples and seven coastal samples (Fig. 4, Tables S9 and S10, Supplementary file 8). The deep-sea samples were taken from four distinct types of deep-sea environments, and the coastal samples were taken from a single type of coastal environment as indicated in the color key of Fig. 4a. We performed a hierarchical clustering analysis to evaluate the relatedness of the oligotype profiles we retrieved. The oligotype profiles show that the diversity of *Woeseiales* bacteria is considerably higher in deep-sea sediments than in coastal ones and that environmental patterns exist. This might reflect strong environmental association at fine taxonomic scale, with many oligotypes strongly overrepresented in either deep-sea or coastal environments. We note that secondary clustering according to geographic origin was also seen for the oligotype profiles retrieved from deep-sea surface sediments, suggesting the existence of geographic effects (Fig. 4a).

We categorized the oligotypes into four distinct groups based on their distribution in the 28 samples we surveyed (Fig. 4b): a group predominantly associated with deep-sea samples (by far the largest group with 180 oligotypes), one associated with coastal samples (34 oligotypes), one including oligotypes present in both deep-sea and coastal samples (58 oligotypes), and a miscellaneous group including oligotypes associated with deep-sea waters, subsurface sediments, or polymetallic nodules (16 oligotypes). Then, we assessed the evolutionary placement of the *Woeseiales* oligotypes to test whether their environmental distribution is consistent with taxon-environment associations inferred from the categorization of the sampling sites of near full-length 16S rRNA gene sequences we recovered from SILVA (Figs. 3, S1 and S2, Table S8). We assigned 180 (out of 288) oligotypes to the lineages we defined in Fig. 3; the placements of the remaining 108 oligotypes were ambiguous and are not further discussed (Table S10).

The evolutionary placement of the oligotypes generally supports environmental distribution patterns we inferred in Fig. 3 with some exceptions (Fig. 4, Table S10). For example, the oligotypes assigned to lineages VII and XIV were well represented in deep-sea samples but were only scantly detected in coastal surface sediment samples. Similarly, the oligotypes assigned to lineages VIa and XII were well represented in the coastal surface sediments but were barely detected in the deep-sea samples. Although none of the oligotypes assigned to lineage Ia were exclusively detected in the coastal samples, a total of 29 oligotypes were detected in both deep-sea and coastal samples, reflecting the presence of members of lineage Ia in coastal environments. Nevertheless, 51 of the 84 oligotypes assigned to lineage Ia were almost exclusively detected in deep-sea samples, which manifests the strong representation of this lineage in deep-sea environments: i.e., oligotypes assigned to lineage Ia accounted on average for 40.2% of the *Woeseiales* 16S rRNA gene sequence tags we recovered from each deep-sea surface sediment sample. More surprisingly, a total of 20 oligotypes assigned to lineage Ib were primarily detected in deep-sea samples, whereas only eight oligotypes assigned to this lineage were predominantly detected in coastal samples; an observation which seems to contrast with the marked preference of lineage Ib for coastal environments we inferred in Fig. 3. However, the 20 oligotypes associated with deep-sea environments accounted, altogether, for a relatively moderate proportion (5.1%) of the *Woeseiales* 16S rRNA gene sequence tags we recovered from the deep-sea surface sediment samples, which could explain the limited detection of members of lineage Ib in the extensive surveying approach (i.e., many sites, shallow sampling of the sites) we used for the analysis shown in Fig. 3. In contrast, the eight oligotypes prevalent in the coastal surface sediment samples accounted for 22.2% of the sequence tags we retrieved from these samples. Likewise, distinct sets of oligotypes associated with either deep-sea or coastal samples, accounted for the presence of lineages V, X, and XI in both deep-sea and coastal environments (Fig. 4 and Table S10).

We conclude that at high taxonomic resolution, several taxa (i.e., oligotypes) within *Woeseiales* appear to have a marked association with an environmental category, and especially that taxa prevalent in the deep sea are distinct from those prevailing in coastal seas (Table S10 and Supplementary text 11 for further discussion). We observed varying degrees of association between taxa and environmental categories at lower taxonomic resolution (i.e., phylotypes and lineages), which manifested, in many cases, the existence of subgroups of organisms with distinct environmental distribution patterns within the same lineage. Although strict patterns of associations between *Woeseiales* taxa and environments cannot be firmly established at this stage without surveying *Woeseiales* assemblages at various other geographical sites, we would like to point out that microbial dispersal and dormancy can account for the presence of *Woeseiales* bacteria in environments that do not (or only partially) realize their niche [52–54], which would result in an apparent lack of strict taxa-environment associations. This hypothesis could be tested using measurements of in situ activity and growth rates as enabled, for example, by the development of highly sensitive methodologies for tracing the incorporation of isotopically labeled water into microbial cells [55–57].

### Abundance of *Woeseiales* bacteria in the deep sea

Using CARD–FISH-based microscopic counts, we quantified the abundance of *Woeseiales* bacteria in 40 deep-sea sediment samples collected from 23 globally distributed sites (Fig. 1, Table S3) and in 17 marine water samples collected from four sites located within the LTER HAUSGARTEN in Fram Strait (Supplementary text 12, Table S3). To perform this survey, we designed novel oligonucleotide probes targeting *Woeseiales* 16S rRNA sequences: in silico analysis showed that the novel probes matched 56–81% of the target sequences in SILVA release 132, whereas previously published probes [6], based on SILVA release 117, matched only 5–22% of the target sequences in release 132 (Table S2). Together, our novel probes matched 147 of the 171 sequences that represent the phylotypes we retrieved from the hierarchical clustering of the 994 16S rRNA gene clone sequences (≥1400 bp) assigned to JTB255-MBG in SILVA 128 (Table S8). The 24 sequences that were not matched by a mix of our probes (i.e., both probes have one or more mismatches to these sequences) were placed within eight of the 15 lineages shown in Fig. 3, but only lineage XIV was poorly covered by the probe mix (see Table S8 for detailed target ranges of the probes).

**Fig. 5:**
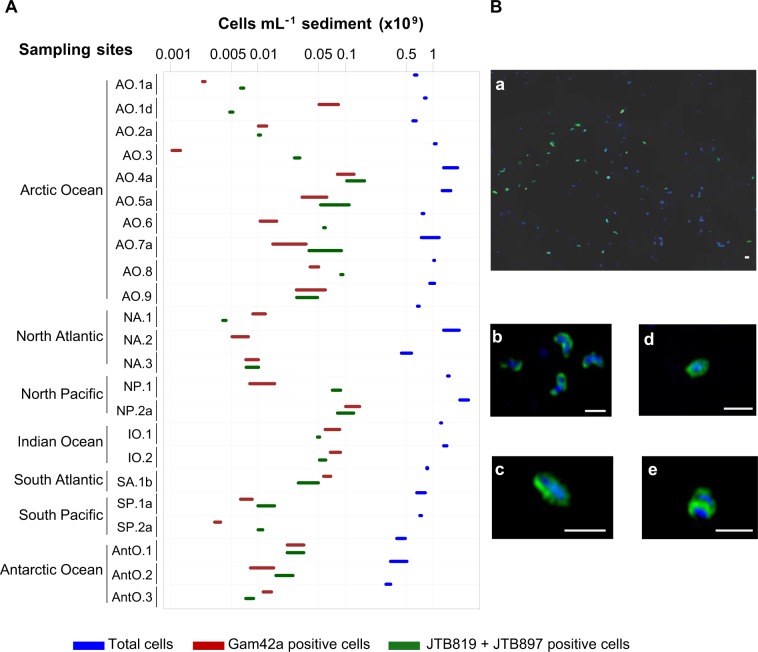
Abundance and morphology of *Woeseiales* cells in deep-sea sediment samples. **a** Plot of cell densities in 23 deep-sea surface sediment samples. Surface sediment refers to sediment collected at the seafloor surface and up to 2 cm below its surface. Total, *Gammaproteobacteria* and *Woeseiales* cell counts are color coded as directly indicated on the figure by the color key. Microscopy-based cell counting of total cells was performed using DAPI as decribed by Schauer et al. [14]. Microscopy-based cell counting of *Gammaproteobacteria* and *Woeseiales* bacteria was performed using catalyzed reporter deposition-fluorescence in situ hybridization (CARD–FISH). *Gammaproteobacteria* were visualized with the probe Gam42a [16]. *Woeseiales* bacteria were visualized with the probes JTB819 and JTB897 (Table S2). The Gam42a probe does not target the members of *Woeseiales*. Colored bars shown on the plot indicate the range of cell density values obtained from technical replicates. Cell density values are detailed in Table S3. Geographic location of the analyzed samples is indicated in Fig. 1 and characteristics of the sampling sites are further described in Table S1. **b** CARD–FISH-based epifluorescence (a) and super-resolution structured illumination (b–e) microscopy images of cells matched by a mix of the oligonucleotide probes JTB819 and JTB897. The sediment sample used to record these pictures was collected in the Artic Ocean (AO.5 site). Images b–e illustrate the different shapes of cells matched by the probes JTB819 and JTB897. DAPI-stained DNA appears in blue and ALEXA488-stained rRNA appears in green. White scale bars indicate a length of 1 µm. Images were captured on a ELYRA PS.1 Zeiss microscope. A 63×/oil plan-apochromat objective was used with a grating of two for the DAPI stain and three for the CARD–FISH stain Alexa488.

We detected *Woeseiales* cells in deep-sea surface sediments (0–2 cm) of all but one site (Fig. 5a, Table S3). The outlier was from the Logatchev hydrothermal vent field located in the North Atlantic Ocean (NA.2 site). We note, however, that *Woeseiales* cells have been detected in other sediment samples collected from (NA. 3 site) or near (NA. 1 site) the Logatchev hydrothermal vent field [14]. We observed in all samples that *Woeseiales* cells occurred singly (i.e., non-aggregated), and displayed several morphologies (Fig. 5b), including cocci (radius ≈ 0.25 µm), rods (radius ≈ 0.2 µm, length ≈ 1.2 µm), and curved rods (radius ≈ 0.2 µm, length ≈ 0.8 µm). This variety of shapes may indicate the existence of several morphologically distinct taxa within *Woeseiales*, but we cannot exclude pleomorphy as an explanation. For example, it was found that cells of the *Woeseiales* type strain (i.e., *W. oceani* XK5) include both rods and curved rods [10].

We found an average *Woeseiales* cell density of 4 × 10^7^ cells cm^−3^ sediment in the top 2 cm of all deep-sea surface sediments surveyed in this study. Cell densities ranged almost over two orders of magnitude, from 4 × 10^6^ cells cm^−3^ sediment (North Atlantic, Logatchev hydrothermal vent field, NA.1 site) to 1 × 10^8^ cells cm^−3^ sediment (continental margin, Fram Strait, AO.4a site) (Fig. 5a, Table S3). Cells of *Woeseiales* bacteria accounted for 1–9% of all cells detected by DAPI staining (5 ± 3%, Table S3), similar to proportions reported from tidal (3–6% [6]) and sublittoral coastal sandy sediments (1–6% [9]). For comparison, *Gammaproteobacteria* accounted for 1–25% of DAPI-stained cells in the surveyed deep-sea sediments with the exception of a single sediment sample (site AO.5c in Fram Strait; 4–5 cm below the seafloor surface) where they accounted for up to 67% of DAPI-stained cells (Table S3 and Supplementary text 13). Our observations are consistent with values previously reported for coastal sediments [6]. Together these results suggest that members of *Woeseiales* constitute a prominent component of benthic bacterial communities on a global scale. In fact, to our knowledge, no other taxonomic order is known to account for such a high proportion of microbial cells in marine sediments, although there is a general lack of reference studies comparing the worldwide abundance of multiple microbial taxa in marine sediments.

An integration of cell numbers across the upper 10 cm of sediments, and extrapolation to the global area of the deep seafloor, suggests a global population of *Woeseiales* bacteria on the order of 5 × 10^26^ ± 2 × 10^17^ cells in deep-sea surface sediments (upper 10 cm). This number seems small relative to the globally estimated 2.4 × 10^28^ *Pelagibacterales* (i.e., SAR11) cells [58]. However, our preliminary figures do not take into account the members of *Woeseiales* that are present in coastal sediments, subsurface sediments, marine waters, and continental environments, indicating that globally, *Woeseiales* could be among the most abundant bacterial orders.

We examined the covariation between cell densities of *Woeseiales* bacteria in marine sediments, and both environmental and spatial variables to evaluate their role as factors predicting the distribution of *Woeseiales*. Based on our limited number of observations, we did not observe any clear macrogeographic pattern in cell densities of *Woeseiales* bacteria. We found a weakly positive relationship between *Woeseiales* cell densities and absolute latitude (Spearman rho = 0.35, *p* = 0.005, Fig. S3, Table S11). The densities of *Woeseiales* cells decreased significantly with increasing water depth (Spearman rho = −0.66, *p* < 0.001, Fig. S3, Table S11), despite outliers at both ends of the sampled water depth range (75–5503 m). Furthermore, we noted that *Woeseiales* cell densities appeared to decrease with increasing sediment depth down to 0.16 m (*R*^2^_GLMM_ = 0.27, *p* < 0.001, Table S12). As the annual peak flux of particulate organic matter to the deep-sea increases with latitude, and deposition of fresh organic matter generally decreases with increasing water and sediment depth [59–63], our results suggest a link between the density of *Woeseiales* bacteria and organic nutrient supply in sediments. This might be further supported by tentative correlations between *Woeseiales* cell densities and both sediment TOC content (Spearman rho = 0.46, *p* < 0.001, Table S11) and surface ocean primary productivity estimates (Spearman rho = 0.62, *p* < 0.001, Table S11). Oxygen partial pressure, which decreases with sediment depth, may be an alternative driver of the abundance of *Woeseiales* bacteria (*R*^2^_GLMM_ = 0.49, *p* < 0.001, Table S12); however, *Woeseiales* cells were absent in only two of the four anoxic sediment samples we surveyed (Tables S1 and S3).

### Genomic traits of *Woeseiales* bacteria in deep-sea surface sediments

We used single-cell genomics and metagenomics to gain further insight into the metabolic potential of *Woeseiales* bacteria inhabiting deep-sea surface sediments. We obtained four partial genomes (Table S13), including the single cell genomes B02 (0.2 Mb) and M06 (1.1 Mb) and the metagenome bins bin1_HGIV (1.6 Mb) and bin2_HGIV (1.8 Mb), from deep-sea surface sediments collected at the LTER HAUSGARTEN in Fram Strait (site AO.5a; see Fig. 1 and Table S1), where *Woeseiales* bacteria were abundant (Fig. 5a, Table S3). The single cell genome B02 is not further discussed here as no information relevant to this study could be recovered from it as indicated by its very small size. The single cell genome M06 was placed within lineage VII (mainly detected in the deep sea) based on the phylogenetic analysis of its 16S rRNA gene sequence (Fig. 3, Table S8). We did not detect a 16S rRNA gene sequence in bin1_HGIV and bin2_HGIV and were therefore not able to directly assign these partial genomes to one of the lineages we defined in Fig. 3. However, we were able to use the concatenated alignments of four ribosomal proteins to investigate the phylogenetic placement of bin1_HGIV and bin2_HGIV relative to the genomes of other members of *Woeseiales* (Fig. S4). In this phylogeny, bin2_HGIV appeared as a close relative of M06, and was tentatively assigned to lineage VII. In contrast, bin1_HGIV formed a singleton node and its placement remained therefore unclear. Yet, bin1_HGIV was placed in a well-supported clade together with M06, bin2_HGIV and the isolate *W. oceani* XK5 (placed in lineage IX) to the exclusion of genomes recovered in previous studies [8, 13] and placed in lineages X and XII (Fig. 3). These genomes recovered from coastal and estuarine sediments contained genomic features suggesting a potential for hydrogen- and sulphur-based chemolithoautotrophy (i.e., Sox gene cluster, Hup-like [NiFe] uptake hydrogenase, RubisCO, and phosphoribulokinase); however, these features were not found in *W. oceani*’s genome [8] or in any of the partial genomes obtained here (estimates of their completeness ranged between 16 and 43%). Definitive confirmation of these features’ absences will only be possible once new closed genomes become available.

**Fig. 6:**
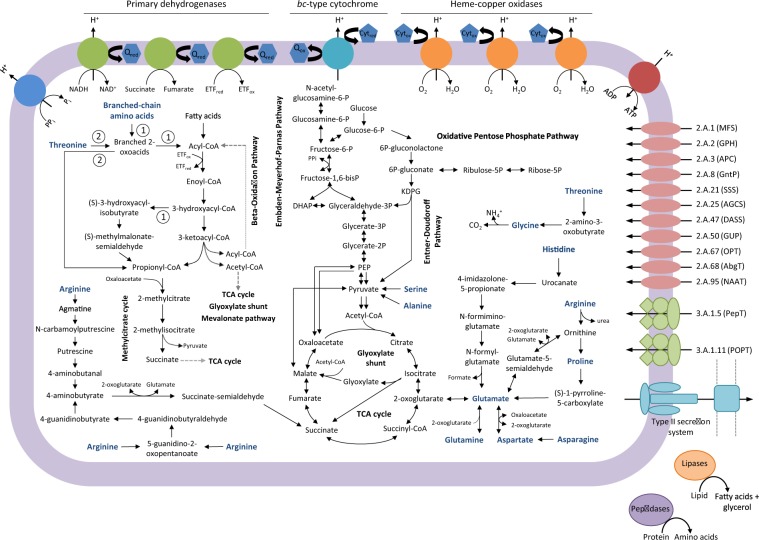
Metabolic map representing catabolic and energy conservation pathways predicted from the genome of *Woeseia oceani* XK5. Genes encoding the predicted pathways are listed in Table S14. The name of compounds serving as potential electron donors and carbon sources was written in blue. Numbers in circles indicate the catabolic route for valine (1) and threonine (2). Transporter types are indicated by their identifiers in the Transporter Classification (TC) database [38]. Full names of the corresponding transporter families are indicated in Table S14. Q_ox_ oxidized respiratory quinone, Q_red_ reduced respiratory quinone, Cyt_ox_ oxidized cytochrome, Cyt_red_ reduced cytochrome.

Considering the phylogenetic relatedness of our new partial genomes to *W. oceani* XK5’s genome (Figs. 3 and  S4), we reconstructed primary catabolic pathways of the XK5 strain from its closed genome sequence (4.1 Mb, 3646 protein-coding genes) to prepare a reference metabolic map (Fig. 6, Table S14) and guide comparisons with our new partial genomes and other members of the *Woeseiales* clade (Table S15). Preliminary investigation of the XK5 strain's physiology and enzymatic activities suggested that it is capable of oxidizing monosaccharides and hydrolyzing lipids and proteins but not polysaccharides [10]; yet none of the reported tests could conclusively show that the strain is capable of using any of the tested compounds as sole source of carbon and energy. Our investigation of *W. oceani*'s gene repertoire suggests that the strain is indeed capable of using monosaccharides, amino acids, and fatty acids as sole carbon and energy sources, and confirms that it has the ability to release amino acids and fatty acids from proteins and lipids, respectively (see Fig. 6, Table S14 and Supplementary text 14 for an in-depth discussion of *W. oceani*’s metabolic potential). We note that *W. oceani*’s primary catabolic pathways mirrors bulk characteristics of the marine biomass, which is rich in lipids and nitrogenous compounds [64].

Considering the proteolytic potential of *W. oceani* XK5 and the preferential biodegradation of proteins over bulk organic matter in both deep-sea and coastal surface sediments [2, 65], we hypothesized that members of *Woeseiales* prevalent in both deep-sea and coastal environments encode a substantial repertoire of putative peptidases. We compared the composition of the peptidase repertoires encoded in the draft genomes recovered here from deep-sea surface sediment with that of *W. oceani* XK5 (lineage IX) and those of the draft genomes previously recovered from coastal (lineage X) and estuarine (lineage XII) sediments to evaluate whether members of distinct lineages of *Woeseiales* have different peptidase repertoires (Table S15). The partial genomes we recovered from deep-sea sediments encoded between 53 and 69 homologs of peptidases (44–64 peptidase homologs per Mb). For comparison, we found that the closed genome of *W. oceani* XK5 encoded 215 putative peptidases (53 peptidase homologs per Mb) and the draft genomes previously recovered from coastal and estuarine sediments 128–309 (52–56 peptidase homologs per Mb). We observed that the repertoires of putative peptidases encoded in all *Woeseiales* genomes we investigated include homologs of peptidases in the M28, S1, and S8 families, which we predict to be responsible for the leucine aminopeptidase (M28) activity and both trypsin and chymotrypsin endopeptidase (S1 and/or S8) activities detected in cultures of *W. oceani* XK5 [10]. These observations support the hypothesis that the members of *Woeseiales* represented both by the partial genomes we recovered here from deep-sea sediments and by those recovered previously from coastal and estuarine sediments [8, 13] are proteolytic. Proteolysis for amino acid uptake may therefore be widely distributed across *Woeseiales*. A total of 10 peptidase families represented in the partial genomes we recovered from deep-sea sediments, and another 30 peptidase families represented in the coastal and estuarine genomes, were not detected in the complete genome of *W. oceani* XK5. This indicates that the repertoire of putative peptidases in members of distinct lineages of *Woeseiales* varies, suggesting functional variability. However, assembling new complete genomes is now needed to establish the environmental and clade specificity of these peptidase repertoires.

A large fraction of the proteinaceous matter in the deep sea resides in refractory high-molecular-weight dissolved organic nitrogen [66] and particulate organic nitrogen [67], which may be mineralized by benthic microorganisms [2, 68, 69] over timescales of hundreds of thousands years [65]. Currently, very little is known about the metabolic machinery and cellular adaptations involved in the degradation of refractory proteins, emphasizing the need to investigate the metabolic and ecological diversity of proteolytic microorganisms.

## Conclusion

The gammaproteobacterial order *Woeseiales* is a globally prominent group of bacteria especially abundant in seafloor surface sediments where it comprises on average 5% of the total number of detectable cells. Lineages within the order *Woeseiales* differ in their environmental distribution and several of these lineages are distinctly associated with deep sea, coastal seas, or continental environments. Yet, taxon-environment associations within *Woeseiales* are more pronounced at higher taxonomic resolution: i.e., lineages often accommodate finer-grained taxa with clearer environmental associations. Furthermore, coastal and especially deep-sea sediments usually host a variety of *Woeseiales* taxa, the co-existence of which suggest they have different ecological functions. For example, whereas individual genomes recovered from coastal and estuarine sediments suggest a potential for chemolithoautotrophy, the coastal strain *W. oceani* XK5 appears to be strictly chemoorganoheterotrophic. Our investigation emphasizes that several relatives of deep-sea and coastal lineages of *Woeseiales* encode homologs of peptidases playing a role in nutrition suggesting that members of *Woeseiales* could be widely involved in the cycling of detrital proteins in marine benthic environments. Yet, the composition of their repertoire of putative peptidases varies, suggesting functional differences among members of *Woeseiales*, which might contribute to their ecological distinctiveness. We expect that systematic investigations of the links between the peptidase repertoire of *Woeseiales* bacteria, their proteolytic activity and preferred environment will shed new light on the cycling of proteinaceous matter in marine environments.

## Supplementary information

Supplementary methods, results and figure legends

Figure S1

Figure S2

Figure S3

Figure S4

Table S1

Table S2

Table S3

Table S4

Table S5

Table S6

Table S7

Table S8

Table S9

Table S10

Table S11

Table S12

Table S13

Table S14

Table S15

Supplementary file 1

Supplementary file 2

Supplementary file 3

Supplementary file 4

Supplementary file 5

Supplementary file 6

Supplementary file 7

Supplementary file 8
