# Supplementary material for: Diversity and metabolism of Woeseiales bacteria, global members of marine sediment communities
Source: ISME J. 2020 Jan 27;14(4):1042–56. doi: 10.1038/s41396-020-0588-4 (PMC7082342; doi:10.1038/s41396-020-0588-4)
Supplement: Supplementary file 1 — Supplementary methods, results and figure legends [file 41396_2020_588_MOESM1_ESM.docx]

Supplementary Information

**Supplementary text 1**: **Design of oligonucleotide probes and CARD-FISH protocol optimization.** Oligonucleotide probes were developed to specifically visualize and count cells of Woeseiales bacteria using CARD-FISH. We searched for conserved oligonucleotide sequences (≥ 17 bp) exclusively present in all full-length 16S rRNA gene sequences (n=755) assigned to Woeseiales (hereafter referred to as test sequences) in SILVA SSUref database release 123 using the ARB probe match tool [1]. Although we did not find an oligonucleotide that was specific for the entire set of test sequences, we found two oligonucleotides (see JTB819 and JTB897 in Table S2) present in 56% and 81% of the test sequences in Silva release 132 (current release). By comparison, published CARD-FISH probes targeting 16S rRNA gene sequences assigned to Woeseiales, i.e. JTB843, JTB1270 and JTB1275 [2], matched only 5, 19 and 22% of the test sequences in Silva release 132, respectively (Table S2). We used a modified version of the probe JTB897 as competitor oligonucleotide (see cJTB897 in Table S2) to minimize hybridization of the probe JTB897 to non-target sequences assigned to the phylum Bacteroidetes (19 049 sequences having one mismatch with the probe sequence), which is usually well represented in marine sediments. Competitor oligonucleotide cJTB897 and horseradish peroxidase-labeled versions of the probes JTB819 and JTB897 were purchased from biomers.net GmbH (Ulm, Germany).

Optimization of the CARD-FISH protocol [3] for visualizing *Woeseiales* cells using a probe mix including the probes JTB819, JTB897 and cJTB897 (mix stoichiometry 1:1:1) was conducted on surface sediments from the LTER HAUSGARTEN in the Fram Strait (PS85/460-4, AO.5a, Table S1). We adjusted the stringency of the hybridization by testing a range of formamide concentrations (0 to 55%) and optimized the cell permeabilization step by varying conditions for the enzymatic digestion of the cell wall of *Woeseiales* cells. The total number of visualized cells as well as their sharpness and brightness was maximal with a formamide concentration of 20% and a permeabilization step consisting of two successive incubations of the polycarbonate filters at 37°C: first with a lysozyme (Sigma-Aldrich, Germany) solution (1 000 kU mL^-1^) applied for 60 min and then with an achromopeptidase (Sigma-Aldrich) solution (90 U mL^-1^) for 30 min.

We validated the specificity of our CARD-FISH assay by performing double hybridizations using our novel probe mix in combination with: i) another set of probes targeting members of *Woeseiales*; i.e. JTB843, JTB1270 and JTB1275 [2]*,* ii) the probe Gam42a and competitor probe cBET42a [4] targeting members of the *Gammaproteobacteria* and iii) the probe CF319a [5] targeting members of the phylum *Bacteroidetes* (probe features are summarized in Table S2). Double-hybridizations with the probes JTB843, JTB1270 and JTB1275 showed that 90-95% of the cells matched by our probe mix were also matched by the set of published probes in both coastal and deep-sea surface sediments. This observation suggested that *Woeseiales* phylotypes matched by our novel probe mix - but missed by previously published probes - accounted for a small fraction of *Woeseiales* cells in the tested samples. Double hybridizations performed with the probe CF319a showed that none of the cells matched by the CF319a probe were detected by our *Woeseiales*-targeting probes. This test suggested that the competitor oligonucleotide cJTB897 might effectively prevent the hybridization of the probe JTB897 to the 16S rRNA of members of the phylum *Bacteroidetes*. Further, cells visualized with our probe mix were not hybridized by the probes Gam42a and cBet42a [4] confirming previous observations suggesting that the probe Gam42a may not match *Woeseiales* sequences [2]. Further, sequence alignments also indicate that the probe Gam42a does not match the 23S rRNA gene sequences of *Woeseia oceani* XK5 and the single-cell amplified genome AG-115_M06 newly obtained in this study.

**Supplementary text 2: Estimating global cell abundances.** Average densities of *Woeseiales* and *Gammaproteobacteria* cells were calculated for five distinct layers of sediment cores (0-1 cm, 1-2 cm, 2-3 cm, 3-5 cm and 5-10 cm from the top of the cores) collected from 23 globally distributed sites (Figure 1, Table S1 and S2). These average cell densities were then used to derive estimates of the overall density of *Woeseiales* bacteria and gammaproteobacteria within the top 10 cm of sediments covering the deep-sea floor. The global abundance of *Woeseiales* cells and *Gammaproteobacteria* cells in deep-sea surface sediments (upper 10 cm) was then extrapolated by multiplying the estimated average density of *Woeseiales* and *Gammaproteobacteria* cells in seafloor surface sediments by the global volume of ocean seafloor sediment (upper 10 cm) below 200 m water depth, i.e. ~3.55 x 10^19^ cm^3^. The volume of the top 10 cm of deep-sea floor sediments was derived from the global area of seafloor below 200 m water depth, i.e. ~357 x 10^6^ km^2^ [6, 7], from which we subtracted the global average area of oceanic ridges (length of ~80 000 km; width of ~20 km) ^[[1]](#footnote-1)^, i.e. ~ 1.6 x 10^6^ km^2^. The standard deviation for global cell number estimates was calculated as the square root of the sum of variances from the different depth layers. The total counts of gammaproteobacterial cells were calculated by summing cell counts obtained with the CARD-FISH probes Gam42a targeting some but not all members of *Gammaproteobacteria* (i.e. Gam42a does not match members of the gammaproteobacterial order *Woeseiales*) and JTB819/JTB897 targeting members of *Woeseiales* (see Supplementary text 1).

**Supplementary text 3: Phylogenetic analyses of 16S rRNA gene sequences.** Accession numbers of 16S rRNA gene sequences used for the inference of phylogenies and their classification in SILVA release 128 are listed in Table S7. For the placement of *Woeseiales* within *Gammaproteobacteria*, we used 591 reference sequences (≥1 400 bp) covering all major, validly named, gammaproteobacterial orders (Table S7). Sequences were aligned with MAFFT v7 [8] using the iterative refinement method L-INS-i [9]. We used the following parameters for alignment: 200PAM/k=2 scoring matrix, a gap opening penalty of 1.53, and an offset value of 0.123. Trimming of unreliable sections of the alignment were performed with BMGE [10] using a minimum block size of three nucleotides. Phylogenies were inferred using a maximum likelihood method as implemented in IQ-TREE [11]. Selection of models of DNA sequence evolution was performed using ModelFinder [12] as implemented in IQ-TREE. Branch support was assessed using 1 000 replicates of ultrafast bootstrap [13] and the Shimodaira–Hasegawa approximate likelihood-ratio test [14].

**Supplementary text 4: Correspondence analysis.** We investigated associations between phylotypes and environmental categories by performing a correspondence analysis (CA) of an environment × phylotype (row × column) matrix (Table S6) in R [15] using the vegan package [16]. To view the association of phylotypes and environmental category scores in more detail, we performed an informal cluster analysis (average linkage) on a matrix containing both the phylotype and environmental category CA scores converted into a Euclidean dissimilarity matrix. This was visualised as a dendrogram using the ape package [17] and nodes grouped using the cutree function (k = 8) were given distinct colors.

**Supplementary text 5: Profiling of Woeseiales oligotypes in marine water and sediment samples.** Profiles of Woeseiales oligotypes were derived from the high-throughput sequencing of 16S rRNA gene amplicons obtained from 34 samples including 26 samples from deep-sea sites and 8 from coastal sites (Figure 1, Table S1). Amplicons were obtained from each of the 34 samples with PCR primers targeting the V3-V4 region of bacterial 16S rRNA gene sequences. Amplicon sequences were newly generated for 26 of the 34 samples whereas those deriving from the other 8 samples were generated in previous studies (Table S1). DNA extraction, PCR conditions, amplicon sequencing and sequence pre-processing for sequences obtained within this study were performed as previously described [18]. Methods for previously published sequences are available in the respective publications (Table S1).

Next, *Woeseiales* 16S sequence tags were extracted from the 16S tag datasets before being grouped into oligotypes [19] using Minimum Entropy Decomposition (MED) v2.1 [20]. To extract *Woeseiales* 16S sequence tags, we first queried the 34 sequence datasets against a database of 190 JTB255 16S rRNA gene clone sequences (Supplementary file 1) using blastn searches (BLAST version 2.2.31+). The 190 sequences in the database were assigned to 58 of the 171 JTB255 phylotypes we generated in this study (Table S4), 12 of which were the most sampled (=sequence rich) of the 171 phylotypes. We filtered out sequence alignments having an e-value higher than 1e-10 in order to exclude spurious hits (Supplementary file 2). To select *Woeseiales* 16S tags among all of the correctly aligned 16S sequence tags, we filtered out: i) sequences sharing less than 95% pairwise sequence identity with their best hit, ii) sequences having an alignment shorter than 400 bp, and iii) sequences having a query alignment start position > 15 and end position < 413 (Supplementary file 3). This procedure recovered a total of 165 436 *Woeseiales* 16S sequence tags from the investigated samples (Table S9).

To group the recovered *Woeseiales* 16S rRNA gene sequence tags into oligotypes, we first padded the sequence tags with gaps as required for MED. Between 0 and 58 gaps were added per sequence; 44 gaps were added in most cases (162 332 out of 165 436). We then performed the oligotyping step using the MED algorithm (Supplementary file 4). Samples totalizing less than 100 *Woeseiales* 16S sequence tags were then excluded from follow-up analyses resulting in the exclusion of six samples (i.e. 28 samples/datasets were kept for subsequent steps). Next, we generated a sample × oligotype (row × column) matrix reporting oligotype sequence counts for each sample and performed a cluster analysis of the investigated *Woeseiales* assemblages based on Bray-Curtis dissimilarity values calculated from the proportions of the oligotypes in each of the 28 oligotype profiles. All analyses were conducted in R [15] using the core distribution of the program (version 3.3.2) and the following packages: we used vegan version 2.4-1 [16] to perform the cluster analysis, iNEXT version 2.0.12 [21] to perform rarefaction analyses and calculate Hill numbers and gplots version 3.0.1 [22] to plot both the dendrogram and heatmap.

**Supplementary text 6: Evaluating the evolutionary placement of the *Woeseiales* oligotypes.** We investigated the evolutionary placement of one representative sequence for each of the *Woeseiales* oligotypes we resolved and assigned these sequences to the phylotypes and lineages we defined based on near full-length 16S rRNA gene sequences (see method paragraph “*Environmental distribution of 16S rRNA gene sequences of Woeseiales bacteria*” in the main text). A list of representative sequences was generated with the tool we used for the oligotyping, i.e. MED v2.1 [20]. The representative sequences were aligned with the near full-length 16S rRNA gene sequences (> 1 400 bp) that served for the inference of the phylogeny shown in Figure 3. To do so, we used the function --addfragments of MAFFT v7 [8] in combination with the original alignment of the near full length 16S sequences (see Supplementary text 3). The addition of the representative sequences to the alignment of near full-length 16S sequences was performed with the following settings: --keeplength option on, the --multipair strategy, a 200PAM/k=2 scoring matrix, a gap opening penalty of 1.53, and an offset value of 0.123. The resulting alignment of short and near full-length 16S rRNA gene sequences was used as input for the evolutionary placement algorithm (EPA) implemented in RAxML v8.2.X [23, 24] together with the phylogeny shown in Figure 3, which served as reference tree. The EPA was executed with the GTRGAMMA model of nucleotide substitution. All possible placements of the representative sequences (between 1 and 7 per sequence) and their likelihood are specified in the .jplace file [25] that we provide as Supplementary file 5. Considering that the likelihood values of all possible placements of a single sequence are only marginally different (Supplementary file 5), we chose a conservative, consensus approach to assign representative sequences to the lineages shown in Figure 3: i.e. a placement was reported only when all placements of a representative sequence were within the same lineage with the allowance of a single incongruent placement (i.e. a placement within a different lineage) for cases where three of more placements were possible.

**Supplementary text 7: Sorting of *Woeseiales* single cells from deep-sea surface sediments.** Two cells were identified as *Woeseiales* bacteria among 498 sorted single cells; an observation, which appears much lower than the counted proportion of *Woeseiales* cells (5 ± 1% of total cell counts) in the original sample. We attempted to improve the recovery of *Woeseiales* cells by sonicating the sediment sample prior to sorting, hypothesizing that the adhesion of *Woeseiales* cells to sediment particles [26] could result in their exclusion during the cell-sorting procedure. Microscopic counts of *Woeseiales* cells in sonicated and non-sonicated sediment samples using CARD-FISH for cell visualization yielded similar cell counts (Supplementary file 6) indicating no improvement in the recovery of *Woeseiales* cells from the sample. We then hypothesized that the standard single cell lysis procedure used at the Bigelow Laboratory Single Cell Genomics Center could be inadequate to release DNA from *Woeseiales* cells based on the observation that hybridization of CARD-FISH probes to *Woeseiales* cells required two consecutive cell wall permeabilization treatments (i.e. lysozyme + achromopeptidase) instead of the more usual lysozyme treatment. Therefore, we attempted to improve the recovery of DNA from the sorted cells using a more drastic cell lysis procedure involving a higher lysis temperature, which was however unsuccessful.

**Supplementary text 8: Phylogenetic analysis of ribosomal proteins.** We investigated the phylogenetic relationships between *Woeseia oceani* XK5 and the metagenome bins and SAGs investigated in this study (Table S13). We searched for shared protein-coding, taxonomic marker genes in the available metagenome bins and SAGs, as several of these draft genomes had either no or only partial rRNA genes (Table S13). The genes encoding the ribosomal proteins S17p, L14p, L16p and L29p were present in all investigated genomes, bins and SAGs (with the exception of SAG B02) and were therefore used for phylogenetic inference. We used *Steroidobacter denitrificans* as outgroup based on our analysis of 16S rRNA gene clone sequences (see main text and Supplementary text 9). Amino acid sequences available for each of the four ribosomal proteins were first aligned with MAFFT v7 [8] using the iterative refinement method L-INS-i [9]. We used the following parameters for the alignment: BLOSUM62 scoring matrix, a gap opening penalty of 1.53, and an offset value of 0.123. Trimming of unreliable sections of the alignments was performed with TCS [27] using default parameters. Trimmed amino acid sequence alignments obtained for the four ribosomal proteins were then concatenated into a supermatrix using catfasta2phyml (https://github.com/nylander/catfasta2phyml). The phylogeny was then inferred using a maximum likelihood method as implemented in IQ-TREE [11]. Selection of models of protein sequence evolution was performed using ModelFinder [12] as implemented in IQ-TREE. Branch support was assessed using 1 000 replicates of ultrafast bootstrap [13] and the Shimodaira–Hasegawa approximate likelihood-ratio test [14].

**Supplementary text 9: Phylogenetic placement of Woeseiales.** The sole cultivated representative of Woeseiales – Woeseia oceani XK5 – has been originally classified within a new family (i.e. Woeseiaceae) assigned to the order Chromatiales [28]. The classification of Woeseiales/Woeseiaceae is however problematic since Chromatiales (as conventionally defined in the Bacterial Nomenclature) appears to be polyphyletic [29, 30], a claim, which was also supported by our analysis (Figure 2, Table S7). Further, Woeseiales/Woeseiaceae did not appear to robustly cluster with any of the lineages typically assigned to Chromatiales, e.g. Chromatiaceae, Ectothiorhodospiraceae and Halothiobacillaceae. We propose that the initial classification of W. oceani XK5 as a member of Chromatiales was the result of a phylogenetic analysis based on a very sparse taxon sample biased towards representatives of Chromatiales [28]. We note that the order Chromatiales as defined in the Genome Taxonomy Database is approximately equivalent to Chromatiaceae in conventional taxonomies.

*Woeseiales/Woeseiaceae* instead formed a supported clade together with 16S rRNA gene clone sequences retrieved from continental environments and including those of *Steroidobacter denitrificans* and *Povalibacter uvarum* (see main text), which are currently assigned to the order *Nevskiales* [31]. With the exception of *S. denitrificans* and *P. uvarum*, *Nevskiales* formed a well-supported monophyletic clade sharing no direct common ancestor with *Woeseiales/Woeseiaceae* in our phylogeny (Figure 2, Table S7). *S. denitrificans* and *P. uvarum* were placed within *Woeseiales* when *Nevskiales* was used as outgroup for *Woeseiales/Woeseiaceae* (Figure 3) further suggesting a placement of *S. denitrificans* and *P. uvarum* outside of *Nevskiales*. In the concatenated protein phylogeny of the Genome Taxonomy DataBase [30], *Woeseiales* and *S. denitrificans* (*P. uvarum*’s genome is not yet available) were also placed together in a well-supported clade outside *bona fide* *Nevskiales*, supporting results of our phylogenetic analysis.

**Supplementary text 10: Phylotype-environment associations.** Information we retrieved about the environmental origin of the 16S rRNA gene clone sequences (≥ 1 400 bp) assigned to *Woeseiales* in SILVA 128 indicated that the large majority of these sequences (904 of 994) originated from a broad variety of benthic environments, as previously reported for other datasets [32–34]. Some of the investigated sequences were however retrieved from marine waters (44 of 994) and continental environments (46 of 994) including inland waters, sediments and soils (Table S6), indicating that members of *Woeseiales* do not exclusively occur in marine benthic environments. Approximately 50% of the sequences originated from deep-sea environments (497 sequences), 39% from coastal environments (390 sequences), 5% from continental environments (46 sequences) and 6% from sources that could not be unambiguously classified (61 sequences). Sequences obtained from deep-sea environments were distributed in 80 phylotypes, those from coastal environments in 113 phylotypes, and those from continental environments in 23 phylotypes. Sequences from unclassified sources belonged to 37 phylotypes (Table S6).

Our correspondence analysis revealed a strong, distinct correspondence (i.e. association) between specific phylotypes and coastal, deep sea, and, to a lesser extent, continental environments (Figure S1 and S2). A total of 60 phylotypes were ordinated closely to the deep-sea centroid, 84 closely to the coastal zone centroid and 9 closely to the continental environment centroid. Sequences retrieved from continental environments are underrepresented in our data matrix (Table S6) relative to sequences retrieved from the deep sea and coastal zones, which could explain that fewer associations between *Woeseiales* phylotypes and the continental environment were observed (Figure S1 and S2). A more systematic surveying of the environmental distribution of *Woeseiales* phylotypes will clarify whether the number of phylotypes that associate with continental areas is currently underestimated and whether the diversity of typically continental *Woeseiales* taxa is lower than that of marine ones.

Some of the phylotypes ordinated closely to the deep sea, coastal zone and continental environment centroids show nevertheless a certain degree of divergence relative to these centroids (Figure S1 and S2). Such phylotypes were detected in at least two types of environments (e.g. deep sea and coastal zone) but were more frequently detected in one environment over the others (Table S6). Further, we observed that the ordination of 15 phylotypes clearly deviated from the deep sea, coastal zone and continental environment centroids; i.e. phylotypes within the grey, red, purple and pink clusters in Figure S2. These phylotypes were scattered in the ordination plots (Figure S1B and S1C) and showed a larger range of CA scores than phylotypes associated with the deep sea, coastal zone and continental environment centroids, respectively (Figure S2). Various sequences within these 15 phylotypes were not assigned to any one of the three environmental categories used in our classification scheme (Table S6) due to the unavailability of the necessary information.

**Supplementary text 11: Profiles of Woeseiales oligotypes in deep-sea and coastal sediments.** We compared profiles of Woeseiales oligotypes retrieved from 7 coastal sediment samples and 21 deep-sea sediment samples obtained from five, geographically distant, sampling sites located within distinct oceanic provinces (Figure 1 and 4, Table S1, S9 and S10). We resolved 288 oligotypes from 149 952 Woeseiales 16S rRNA sequence tags (average length: 427 bp) retrieved from a total of 2 684 975 16S sequence tags (Table S9). Rarefaction analyses of Woeseiales oligotypes retrieved from each sample indicate that, at most investigated sites, the diversity of Woeseiales oligotypes, represented by its effective number [35], was adequately sampled (Supplementary file 8). A hierarchical clustering analysis of Woeseiales oligotype profiles grouped the profiles derived from deep-sea and coastal samples into two distinct clusters (Figure 4). Oligotype profiles derived from deep-sea samples were further grouped into four distinct clusters; profiles within each of these clusters being derived from a distinct environment: seafloor surface sediments, subsurface sediments, polymetallic nodules and water column, respectively. Further, oligotype profiles derived from deep-sea benthic environments (seafloor surface and subsurface sediments, polymetallic nodules) were grouped together to the exclusion of oligotype profiles derived from deep-water samples. In conclusion, our results indicate that the assemblages of Woeseiales bacteria in deep-sea samples recovered from the Arctic, Indian and South Pacific Oceans are distinct from those in coastal sediment samples collected from the German bight and Western Australia, further hinting at possible taxa-environment associations as discussed below for deep-sea and coastal surface sediments (see Table S10 for supporting data).

A total of 246 oligotypes were detected in deep-sea surface sediment samples and 145 of them were not detected in the coastal sediment samples we investigated. These 145 oligotypes accounted, on average, for 52% (range for individual oligotypes: <0.01-5.65%) of the *Woeseiales* 16S rRNA gene sequence tags retrieved from each deep-sea surface sediment sample. Twenty-four of these 145 oligotypes were detected in all deep-sea surface sediment samples and accounted on average for 24.5% (range for individual oligotypes: 0.08-5.65%) of the *Woeseiales* 16S tags recovered from each deep-sea surface sediment sample. Three of the 24 oligotypes are among those that are most represented in the deep-sea surface sediment samples: i.e. rank 1, 2 and 6 of 246 based on the proportion of reads assigned to the oligotypes. Each of the 24 oligotypes was detected in multiple samples (i.e. median 7 of 11 samples) recovered from other deep-sea environments, i.e. subsurface sediments, polymetallic nodules and deep waters.

A total of 128 oligotypes were detected in the coastal sediment samples and 27 of them were not detected in any the deep-sea surface sediment samples. These 27 oligotypes accounted, on average, for 49% (range for individual oligotypes: <0.01-7.53%) of the JTB255 16S rRNA gene sequence tags retrieved from each coastal sediment sample. Seven of the 27 oligotypes were present in all coastal sediment samples and accounted, on average for 24.5% (range for individual oligotypes: 1.53-7.53%) of the *Woeseiales* 16S tags recovered from each coastal sediment sample. Four of the seven oligotypes are among those that are most represented in the coastal sediment samples: i.e. rank 2, 3, 9 and 10 of 128 based on the proportion of reads assigned to the oligotypes. Only one of these seven oligotypes was detected in a sample recovered from the deep-sea (i.e. a subsurface sediment sample where the oligotype accounted for 0.04% of the *Woeseiales* reads recovered from the sample).

A total of 101 oligotypes were detected in both deep-sea and coastal surface sediment samples. On average, these oligotypes accounted for 48% of the *Woeseiales* reads recovered from each deep-sea surface sediment sample (range for individual oligotypes: <0.01-4.21%) and for 51% of those recovered from each coastal sediment sample (range for individual oligotypes: <0.01-9.20%). Twenty-two of these oligotypes were detected in all deep-sea surface sediment samples and accounted, on average, for 29% of the *Woeseiales* reads recovered from each of these samples (range for individual oligotypes: 0.26-4.21%; 11 oligotypes above 1%). These 22 oligotypes accounted only for 4% of the reads retrieved from each coastal sediment sample (range for individual oligotypes: <0.01-1.30%; 1 oligotype above 1%). Fifteen of these 22 oligotypes were only marginally detected in coastal sediment samples (i.e. each was detected in max. 3 samples; range for individual oligotypes: <0.01-0.17%) and none of them was clearly more represented in coastal sediments than in deep-sea surface sediments. Another six of the 101 oligotypes were detected in all coastal sediment samples and accounted, on average, for 14% of the *Woeseiales* reads recovered from each coastal sample (range for individual oligotypes: 0.36-5.69%; five oligotypes above 1%). These six oligotypes accounted only for 1.5% of the reads recovered from each deep-sea surface samples (range for individual oligotypes: <0.01%-1.24%; 1 oligotype above 1%). Four of these six oligotypes were only marginally detected in deep-sea surface sediment samples (i.e. each was detected in max. 4 samples; range for individual oligotypes: <0.01-0.21%) and were not represented in any of the oligotype profiles we obtained from other deep-sea samples. None of these six oligotypes appeared to be more represented in the deep-sea than in the coastal sediments we analysed.

In conclusion, at least 50 of the 288 *Woeseiales* oligotypes appeared to have marked preferences for the deep-sea (39 oligotypes) and coastal (11 oligotypes) samples we investigated, which further points to taxa-environment associations as suggested by our analyses of *Woeseiales* phylotypes (Figure S1 and S2) and lineages (Figure 3). We note that 29 of the 39 oligotypes that had marked preferences for deep-sea environments were unambiguously placed within some of the *Woeseiales* lineages defined in Figure 3: 17 were assigned to lineage Ia, one to lineage Ib, three to lineage V, three to lineage VII, one to lineage X and four to lineage XI (Table S10). Further, we note that eight of the 11 oligotypes that had marked preferences for coastal surface sediments were also unambiguously placed within some of the *Woeseiales* lineages: one was assigned to lineage Ia, four to lineage Ib, one to lineage III, one to lineage V and one to lineage VIa (Table S10). The evolutionary placement of the 288 oligotypes we resolved is further discussed in the main text (see “*Environmental preferences of taxa within the order Woeseiales”* in the Results and Discussion section).

**Supplementary text 12: Abundance of *Woeseiales* bacteria in marine waters.** Little is known about the occurrence of *Woeseiales* bacteria and their DNA sequences in the marine water column. Investigation of the environmental origin of 16S rRNA gene clone sequences in SILVA 128 showed that *ca*. 5% of the sequences (≥ 1 400 bp) assigned to *Woeseiales* were retrieved from marine water samples (Table S6) and the profiling of *Woeseiales* oligotypes in Antarctic Ocean waters further confirmed the presence of these organisms in the marine water column (Figure 4, Table S10). We were able to detect cells in waters from the LTER HAUSGARTEN, Fram Strait, but at much lower absolute and relative abundances than in sediments (Table S3). The members of *Woeseiales* accounted for <0.1% of all cells in surface waters (2 × 10^2^ - 4 × 10^3^ cells cm^-3^) and up to 2% of all cells in meso- and bathypelagic waters (2 × 10^2^ - 3 × 10^3^ cells cm^-3^). These figures are currently the only data available for marine waters, hence additional surveys are necessary to evaluate global trends of *Woeseiales* cell counts through the water column.

**Supplementary text 13: Global abundance of the members of Gammaproteobacteria in deep-sea sediments.** Densities of gammaproteobacterial cells ranged from 1.4 × 10^6^ cells cm^-3^ sediment in a sample obtained from the Central Arctic Ocean seafloor (AO.3) to 1.7 × 10^8^ cells cm^-3^ sediments in a sample obtained from the South Atlantic Ocean (SA.1a) (Figure 1 and 5A, Table S1 and S2). The global population of gammaproteobacterial cells in deep-sea surface sediments (top 10 cm) was estimated in the order of 1 × 10^27^ ± 2 × 10^17^ cells. For comparison, the global population of Woeseiales cells in deep-sea surface sediments (top 10 cm) was estimated in the order of 5 × 10^26^ ± 2 × 10^17^ cells (see main text).

**Supplementary text 14: Metabolic pathways of Woeseia oceani XK5.** The gene repertoire of W. oceani XK5 (hereafter referred to as XK5) was investigated to reconstruct metabolic pathways that this organism may use to conserve energy and oxidize organic substrates (Figure 6, Table S14). In agreement with its ability to grow aerobically, XK5’s genome encodes all components of an aerobic electron transport chain including complex I to V. XK5’s genome notably encodes three distinct terminal oxidases, all of which are heme-copper oxidases (HCOs) including two class A and one class C enzymes as indicated by a publicly available HCO classifier [36]. Class C HCOs include the cbb_3_-type cytochrome c oxidases, some members of which are known to have a high affinity for oxygen and play roles in microaerophilic growth [37, 38]. A previous report also identified components of a truncated denitrification pathway in the gene repertoire of the XK5 strain as alternative respiratory pathway [34]. Components of this truncated pathway include a homolog of the periplasmic nitrite reductase NirS (locus tag: BA177_05470) and two homologs of the membrane-bound nitric oxide reductase NorB (locus tags: BA177_16390 and BA177_17500). Although this pathway suggests the ability to produce nitrous oxide from the dissimilation of nitrite, it is currently unclear whether it operates under (sub)oxic or anoxic conditions. The XK5 strain was reported to be capable of growth under anoxic conditions [28]; yet the metabolism that enabled its growth in the reported experiment remains unclear.

The genome of XK5 encodes homologs of all enzymes of the Embden-Meyerhof-Parnas (EMP) pathway and Entner-Doudoroff (ED) pathway indicating that the strain has the potential to oxidize intracellular glucose to pyruvate via two distinct routes. Genes encoding a homolog of pyruvate dehydrogenase, all enzymes of the tricarboxylic acid cycle and several anaplerotic enzymes are also present in the gene repertoire of XK5 suggesting that intracellular glucose (and intermediates of its degradation) can be fully oxidized to CO_2_. The XK5 strain was reported to be capable of oxidizing the glycolytic intermediate fructose-6-phosphate but not glucose and glucose-6-phosphate based on the corresponding assays in the Biolog GEN III microplate system [28]. This observation suggests that XK5 may have the ability to take up fructose-6-phosphate but not glucose and glucose-6-phosphate; yet the adequacy of the Biolog GEN III microplate system for the phenotyping of XK5 has not been clearly established and further testing is required before ruling out possible carbon and energy sources. The gene repertoire of XK5 encodes multiple homologs of secondary active transporters representing 11 distinct transporter families involved in the uptake of organic substrates including saccharides but also organic acids and amino acids (Figure 6, Table S14). In contrast, the genome of XK5 encodes only few homologs of the high-affinity, ABC-type, primary active transporters involved in the uptake of organic substrates and no homolog of the phosphotransferase systems, which play important roles in the uptake of saccharides in other gammaproteobacteria, notably members of the *Enterobacteriaceae*. It was previously reported that the genome of *W. oceani* XK5 encodes only 19 homologs of glycoside hydrolases (GHs) supporting the idea that *W. oceani* XK5 is unlikely to use complex polysaccharides [34]. This is emphasized by the fact that 11 of the GH homologs in *W. oceani*’s genome are related to peptidoglycan lytic enzymes (i.e. the GH families GH23, GH73 and GH103), which are usually involved in cell wall maintenance [39]. We note here that the genome of the marine gammaproteobacterium *Saccharophagus degradans* 2-40 [40] – a highly versatile polysaccharide degrader – and *Alteromonas macleodii* ATCC 27126 [41] – a copiotrophic generalist – encode a total of 139 and 41 GH homologs, respectively (as reported in CAZy). For comparison the proteolytic bacteria *Idiomarina loihiensis* [42] and *Pseudoalteromonas haloplanktis* [43] encodes 11 and 18 GH families, respectively.

The genome of XK5 encodes homologs for all enzymes involved in the catabolic pathways of at least 13 of the proteinogenic amino acids including glycine, alanine, valine, isoleucine, serine, threonine, asparagine, aspartate, glutamine, glutamate, proline, histidine and arginine (Figure 6, Table S14). The gene repertoire of XK5 also encodes a variety of proteins that are homologs of both amino acid and peptide uptake transporters, suggesting that XK5 is able to take up and oxidize amino acids. Most remarkably, XK5 appears to possess no less than four pathways for the catabolism of arginine (i.e. two variants of the arginase pathway, the arginine decarboxylase/agmatine deiminase pathway and the arginine:pyruvate transaminase pathway) suggesting that arginine could play an important role in the biology and ecology of XK5. A similar versatility in the catabolism of arginine has been reported in other gammaproteobacteria, especially in the genus *Pseudomonas*: e.g. the proteolytic strain *P. aeruginosa* PAO1 also possesses four distinct pathways for the catabolism of arginine, the presence of which critically influences its lifestyle and activity [44-46]. These observations are in agreement with the growth of XK5 on a peptone and yeast extract-based medium (i.e. 2216E agar) and its ability to hydrolyze gelatin and produce both aminopeptidases and endopeptidases [28]. This is further supported by the observation that the genome of *W. oceani* XK5 encodes up to 222 putative peptidases [34]. For comparisons, the proteolytic marine gammaproteobacteria *A. macleodii* ATCC 27126, *Idiomarina loihiensis* L2-TR(T) [42] and *Pseudoalteromonas haloplanktis* TAC125 [43] encode a total of 270, 101 and 208 peptidase homologs, respectively [47]. The versatile saccharolytic bacterium *S. degradens* 2-40 encodes only few putative peptidases (130) in its relatively large genome (5.06 Mb) considering that the genome of the proteolytic *I. loihensis* (2.8 Mb) encodes nearly as many peptidase homologs.

The gene repertoire of XK5 encodes multiple homologs of each enzyme of the fatty acid β-oxidation pathway (e.g. up to nine homologs of the acyl-CoA dehydrogenase), the alpha and beta subunits of the electron transfer flavoprotein (ETF) and a homolog of the ETF:ubiquinone oxidoreductase suggesting that XK5 has the potential to conserve energy from the oxidation of fatty acids. XK5’s genome also encodes homologs of the glyoxylate shunt enzymes suggesting it is able to use fatty acids as sole carbon source. There is currently no direct experimental confirmation of XK5’s ability to use fatty acids as sole carbon and energy source, yet enzymatic tests performed with the API ZYM kit have indicated that the strain synthesizes a butyrate esterase lipase, a caprylate esterase lipase and a myristate lipase [27], which appears consistent with the ability of utilizing fatty acids (see main text for a summary of the putative hydrolases, including lipases, produced by XK5). We observed that *W. oceani*’s genome encodes at least 10 homologs of lipases (including GDSL-like lipases, Patatin-like phospholipases and PLD-like phospholipases).

**Supplementary References**

1. Ludwig W, Strunk O, Westram R, Richter L, Meier H, Yadhukumar A, et al. ARB: A software environment for sequence data. *Nucleic Acids Res* 2004; **32**: 1363–1371.

2. Dyksma S, Bischof K, Fuchs BM, Hoffmann K, Meier D, Meyerdierks A, et al. Ubiquitous *Gammaproteobacteria* dominate dark carbon fixation in coastal sediments. *ISME J* 2016; **10**: 1939–1953.

3. Ishii K, Mußmann M, MacGregor BJ, Amann R. An improved fluorescence *in situ* hybridization protocol for the identification of bacteria and archaea in marine sediments. *FEMS Microbiol Ecol* 2004; **50**: 203–212.

4. Manz W, Amann R, Ludwig W, Wagner M, Schleifer KH. Phylogenetic oligodeoxynucleotide probes for the major subclasses of *Proteobacteria*: problems and solutions. *Syst Appl Microbiol* 1992; **15**: 593–600.

5. Manz W, Amann R, Ludwig W, Vancanneyt M, Schleifer KH. Application of a suite of 16S rRNA-specific oligonucleotide probes designed to investigate bacteria of the phylum cytophaga-flavobacter-bacteroides in the natural environment. *Microbiology* 1996; **142**: 1097–1106.

6. Costello MJ, Coll M, Danovaro R, Halpin P, Ojaveer H, Miloslavich P. A Census of marine biodiversity knowledge, resources, and future challenges. *PLoS One* 2010; **5**: e12110.

7. de Lavergne C, Madec G, Capet X, Maze G, Roquet F. Getting to the bottom of the ocean. *Nat Geosci* 2016; **9**: 857–858.

8. Katoh K, Standley DM. MAFFT multiple sequence alignment software version 7 : Improvements in performance and usability. *Mol Biol Evol* 2013; **30**: 772–780.

9. Katoh K, Toh H. Recent developments in the MAFFT multiple sequence alignment program. *Brief Bioinformtics* 2008; **9**: 81–92.

10. Criscuolo A, Gribaldo S. BMGE (Block Mapping and Gathering with Entropy): A new software for selection of phylogenetic informative regions from multiple sequence alignments. *BMC Evol Biol* 2010; **10**: 210.

11. Nguyen L, Schmidt HA, Haeseler A Von, Minh BQ. IQ-TREE : A fast and effective stochastic algorithm for estimating maximum-likelihood phylogenies. *Mol Biol Evol* 2014; **32**: 268–274.

12. Kalyaanamoorthy S, Minh BQ, Wong TKF, Von Haeseler A, Jermiin LS. ModelFinder: Fast model selection for accurate phylogenetic estimates. *Nat Methods* 2017; **14**: 587–589.

13. Minh BQ, Nguyen MAT, Von Haeseler A. Ultrafast approximation for phylogenetic bootstrap. *Mol Biol Evol* 2013; **30**: 1188–1195.

14. Guindon S, Dufayard JF, Lefort V, Anisimova M, Hordijk W, Gascuel O. New algorithms and methods to estimate maximum-likelihood phylogenies: Assessing the performance of PhyML 3.0. *Syst Biol* 2010; **59**: 307–321.

15. R core team. R: A language and environment for statistical computing. *R Found Stat Comput Vienna, Austria* 2017.

16. Oksanen J, Blanchet FG, Friendly M, Kindt R, Legendre P, Mcglinn D, et al. vegan: Community ecology package. *R Packag version 24-4* 2017.

17. Paradis E, Claude J, Strimmer K. APE: Analyses of phylogenetics and evolution in R language. *Bioinformatics* 2004; **20**: 289–290.

18. Hoffmann K, Hassenrück C, Salman-Carvalho V, Holtappels M, Bienhold C. Response of bacterial communities to different detritus compositions in arctic deep-sea sediments. *Front Microbiol* 2017; **8**: 266.

19. Eren AM, Maignien L, Sul WJ, Murphy LG, Grim SL, Morrison HG, et al. Oligotyping: Differentiating between closely related microbial taxa using 16S rRNA gene data. *Methods Ecol Evol* 2013; **4**: 1111–1119.

20. Eren AM, Morrison HG, Lescault PJ, Reveillaud J, Vineis JH, Sogin ML. Minimum entropy decomposition : Unsupervised oligotyping for sensitive partitioning of high- throughput marker gene sequences. *ISME J* 2015; **9**: 968–979.

21. Hsieh TC, Ma KH, Chao A. iNEXT: an R package for rarefaction and extrapolation of species diversity (Hill numbers). *Methods Ecol Evol* 2016; **7**: 1451–1456.

22. Warnes AGR, Bolker B, Bonebakker L, Huber W, Liaw A, Lumley T, et al. Package gplots. 2016. , 1–68

23. Berger SA, Krompass D, Stamatakis A. Performance, accuracy, and web server for evolutionary placement of short sequence reads under maximum likelihood. *Syst Biol* 2011; **60**: 291–302.

24. Stamatakis A. RAxML version 8: A tool for phylogenetic analysis and post-analysis of large phylogenies. *Bioinformatics* 2014; **30**: 1312–1313.

25. Matsen FA, Hoffman NG, Gallagher A, Stamatakis A. A format for phylogenetic placements. *PLoS One* 2012; **7**: e31009.

26. Probandt D, Eickhorst T, Ellrott A, Amann R, Knittel K. Microbial life on a sand grain: From bulk sediment to single grains. *ISME J* 2018; **12**: 623–633.

27. Chang JM, Di Tommaso P, Notredame C. TCS: A new multiple sequence alignment reliability measure to estimate alignment accuracy and improve phylogenetic tree reconstruction. *Mol Biol Evol* 2014; **31**: 1625–1637.

28. Du ZJ, Wang ZJ, Zhao JX, Chen GJ. *Woeseia oceani* gen. nov., sp. nov., a chemoheterotrophic member of the order *Chromatiales*, and proposal of *Woeseiaceae* fam. nov. *Int J Syst Evol Microbiol* 2016; **66**: 107–112.

29. Williams KP, Gillespie JJ, Sobral BWS, Nordberg EK, Snyder EE, Shallom JM, et al. Phylogeny of *Gammaproteobacteria*. *J Bacteriol* 2010; **192**: 2305–2314.

30. Parks DH, Chuvochina M, Waite DW, Rinke C, Skarshewski A, Chaumeil P-A, et al. A standardized bacterial taxonomy based on genome phylogeny substantially revises the tree of life. *Nat Biotechnol* 2018; **36**: 996–1004.

31. Naushad S, Adeolu M, Wong S, Sohail M, Schellhorn HE, Gupta RS. A phylogenomic and molecular marker based taxonomic framework for the order *Xanthomonadales*: Proposal to transfer the families *Algiphilaceae* and *Solimonadaceae* to the order *Nevskiales* ord. nov. and to create a new family within the order *Xanthomonadales*, the family *Rhodanobacteraceae* fam. nov., containing the genus *Rhodanobacter* and its closest relatives. *Antonie Van Leeuwenhoek* 2015; **107**: 467–485.

32. Schauer R, Bienhold C, Ramette A, Harder J. Bacterial diversity and biogeography in deep-sea surface sediments of the South Atlantic Ocean. *ISME J* 2010; **4**: 159–170.

33. Bienhold C, Zinger L, Boetius A, Ramette A. Diversity and biogeography of bathyal and abyssal seafloor bacteria. *PLoS One* 2016; **11**: e0148016.

34. Mußmann M, Pjevac P, Krüger K, Dyksma S. Genomic repertoire of the *Woeseiaceae*/JTB255, cosmopolitan and abundant core members of microbial communities in marine sediments. *ISME J* 2017; **11**: 1276–1281.

35. Chao A, Gotelli NJ, Hsieh TC, Sander EL, Ma KH, Colwell RK, et al. Rarefaction and extrapolation with Hill numbers: A framework for sampling and estimation in species diversity studies. *Ecol Monogr* 2014; **84**: 45–67.

36. Sousa FL, Alves RJ, Pereira-Leal JB, Teixeira M, Pereira MM. A bioinformatics classifier and database for Heme-Copper oxygen reductases. *PLoS One* 2011; **6:** e19117.

37. Ekici S, Pawlik G, Lohmeyer E, Koch HG, Daldal F. Biogenesis of *cbb*3-type cytochrome *c* oxidase in *Rhodobacter capsulatus*. *Biochim Biophys Acta - Bioenerg* 2012; **1817**: 898-910.

38. Hirai T, Osamura T, Ishii M, Arai H. Expression of multiple *cbb*3 cytochrome *c* oxidase isoforms by combinations of multiple isosubunits in *Pseudomonas aeruginosa*. *Proc Natl Acad Sci USA* 2016; **113**: 12815–12819.

39. Park JT, Uehara T. How bacteria consume their own exoskeletons (turnover and recycling of cell wall peptidoglycan). *Microbiol Mol Biol Rev* 2008; **72**: 211–227.

40. Weiner RM, Taylor LE, Henrissat B, Hauser L, Land M, Coutinho PM, et al. Complete genome sequence of the complex carbohydrate-degrading marine bacterium, *Saccharophagus degradans* strain 2-40T. *PLoS Genet* 2008; **4**: e1000087.

41. Vandecandelaere I, Nercessian O, Segaert E, Achouak W, Mollica A, Faimali M, et al. *Alteromonas genovensis* sp. nov., isolated from a marine electroactive biofilm and emended description of *Alteromonas macleodii* Baumann et al. 1972 (Approved Lists 1980). *Int J Syst Evol Microbiol* 2008; **58**: 2589–2596

42. Hou S, Saw JH, Lee KS, Freitas TA, Belisle C, Kawarabayasi Y, et al. Genome sequence of the deep-sea -proteobacterium *Idiomarina loihiensis* reveals amino acid fermentation as a source of carbon and energy. *Proc Natl Acad Sci USA* 2004; **101**: 18036–18041

43. Médigue C, Krin E, Pascal G, Barbe V, Bernsel A, Bertin PN, et al. Coping with cold: The genome of the versatile marine Antarctica bacterium *Pseudoalteromonas haloplanktis* TAC125. *Genome Res* 2005; **15**: 1325–1335

44. Palmer KL, Mashburn LM, Singh PK, Whiteley M. Cystic fibrosis sputum supports growth and cues key aspects of *Pseudomonas aeruginosa* physiology. *J Bacteriol* 2005; **187**: 5267–5277.

45. Bernier SP, Ha DG, Khan W, Merritt JH, O’Toole GA. Modulation of *Pseudomonas aeruginosa* surface-associated group behaviors by individual amino acids through c-di-GMP signaling. *Res Microbiol* 2011; **162**: 680–688.

46. Everett J, Turner K, Cai Q, Gordon V, Whiteley M, Rumbaugh K. Arginine is a critical substrate for the pathogenesis of *Pseudomonas aeruginosa* in burn wound infections. *mBio* 2017; **8:** e02160-16.

47. Rawlings ND, Barrett AJ, Thomas PD, Huang X, Bateman A, Finn RD. The MEROPS database of proteolytic enzymes, their substrates and inhibitors in 2017 and a comparison with peptidases in the PANTHER database. *Nucleic Acids Res* 2018; **46**: D624–D632

48. Seiter K, Hensen C, Schröter J, Zabel M. Organic carbon content in surface sediments - Defining regional provinces. *Deep Res Part I Oceanogr Res Pap* 2004; **51**: 2001–2026.

**Supplementary figure legends**

**Figure S1**. Correspondence analysis (CA) biplots depicting associations between 171 phylotypes and four environmental categories. The phylotypes comprised 994 16S rRNA gene sequences assigned to the gammaproteobacterial order *Woeseiales*. The four environmental categories were the deep-sea, coastal zone, continental and unclassified environments. The data matrix analyzed is shown in Table S6. The CA solution is shown in three biplots (A-C) displaying all combinations of the first three axes (A: 1 and 2, B: 1 and 3, C: 2 and 3). The sum of the eigenvalues of these axes (1: 0.7212, 2: 0.5167, and 3: 0.3557) account for nearly all the inertia in the solution (1.594). Phylotypes are visualized as blue diamonds with a semi-transparent fill to show overlap density. Environmental category centroids are shown as red-filled circles and labeled. Note, due to the purely exploratory nature of this analysis and the ad hoc collection of samples grouped into environmental categories (rather than an analysis of a true gradient of sites), no correction was applied to the original data matrix, and the ordination is bound by a compositional simplex.

**Figure S2**. Dendrogram depicting the hierarchical clustering patterns of scores calculated in the correspondence analysis (CA) of a data matrix including 171 phylotypes and four environmental categories. The phylotypes grouped 994 16S rRNA gene sequences assigned to gammaproteobacterial order *Woeseiales* (Table S6). The four environmental categories grouped environments from the deep-sea, coastal zones, continents and unclassified environments, respectively. Corresponding CA biplots are shown in Figure S1. CA scores were converted into an Euclidean distance matrix before applying an average linkage clustering. The dendrogram was visualized with the ‘ape’ package [17] and the eight coloured clusters defined with the cutree function (k=8).

**Figure S3**. Relationship between the density of cells visualized with a mix of the CARD-FISH probes JTB819 and JTB897 (Table S2) in sediments samples and: (**A**) the bottom depth of the ocean at the sediment sampling sites, (**B**) the absolute latitude of the sampling locations, (**C**) the total organic carbon (TOC) content of the sediment samples and (**D**) primary production values at the sampling locations. Primary production values used for the analysis were reported in Seiter *et al*. [48]. Cell densities and primary production values are represented as the log_10_-transform of the original data. Each circle represents the average density of *Woeseiales* cells in a sediment sample, which was calculated as the arithmetic mean of duplicate cell counts obtained for that sample. Error bars represent range between lowest and highest replicate cell counts. Trend lines are based on linear regression and 95% confidence interval (see Table S11 for regression and correlation statistics).

**Figure S4**. Maximum likelihood phylogeny of draft genomes investigated in this study. Characteristics of the draft genomes are shown in Table S13. The genome of *Woeseia oceani* XK5 was used as reference for the gammaproteobacterial order *Woeseiales* and the genome of *Steroidobacter denitrificans* F5 was used as outgroup. The phylogeny is based on the concatenated alignments of the amino acid sequences of four ribosomal proteins; i.e. S17p, L14p, L16p and L29p. The phylogeny was inferred using IQ-TREE [11]. Inference was based on the LG model of amino acid substitution and a model of rate heterogeneity across sites that included four rate categories following a gamma distribution (LG + G4). Scale bar indicates the number of amino acid substitutions per site. Branch support was evaluated using 1000 replicates of ultra-fast bootstrap (UFBoot) and Shimodaira-Hasegawa approximate likelihood-ratio test (SH-aLRT). Branch support was only indicated for those branches that had a support ≥70% using both methods. Lineage numbers indicated in parentheses on the phylogeny next to the draft genome’s name refer to 16S rRNA gene sequence clusters to which the genomes were assigned (see Figure 3).

1. 1<https://www.britannica.com/science/oceanic-ridge>, 21.04.2017, 17 o`clock. [↑](#footnote-ref-1)
