# Supplementary figures and images for: Diversity and metabolism of Woeseiales bacteria, global members of marine sediment communities
Source: ISME J. 2020 Jan 27;14(4):1042–56. doi: 10.1038/s41396-020-0588-4 (PMC7082342; doi:10.1038/s41396-020-0588-4)

Figure S1

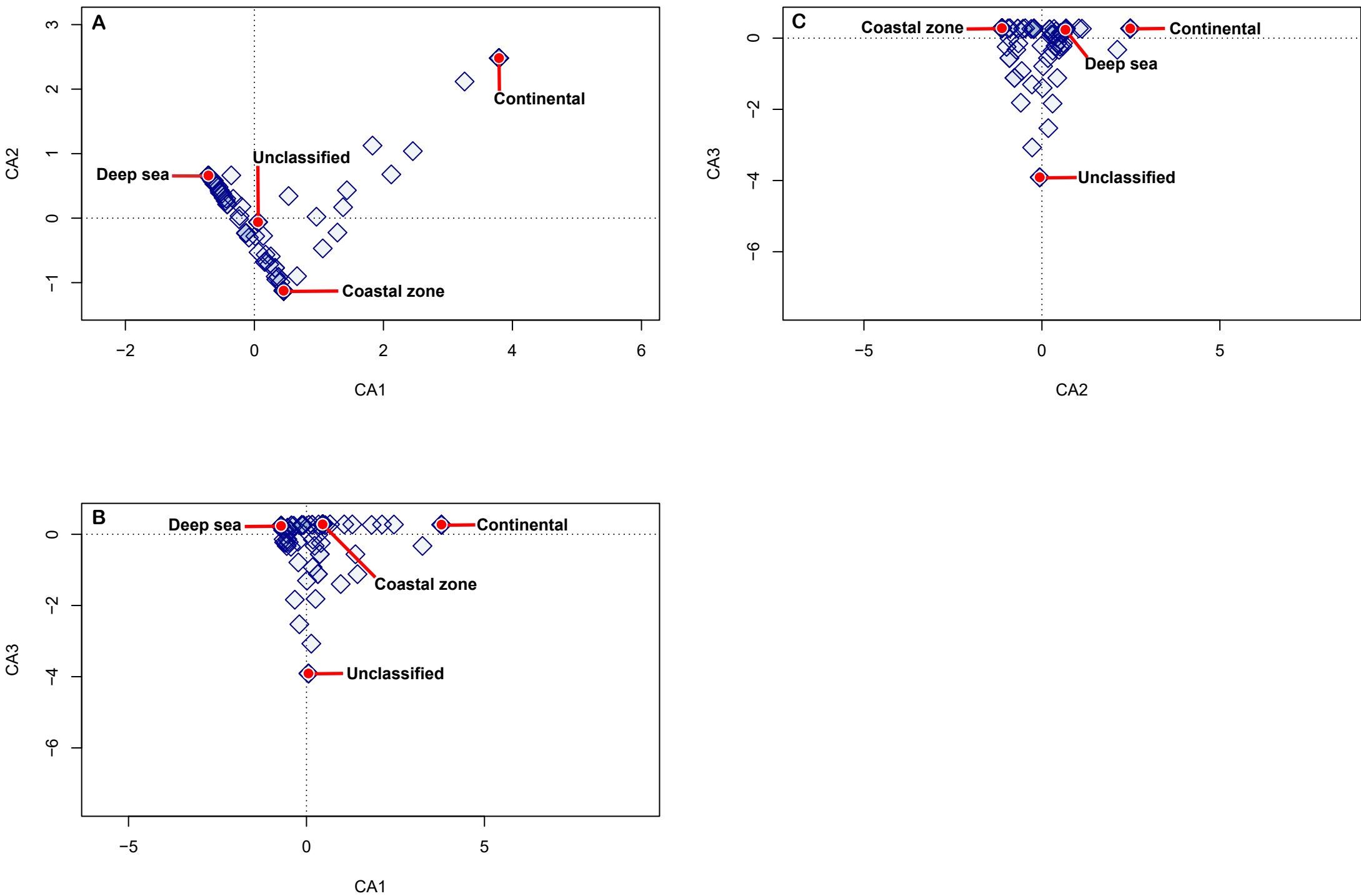

Supplement: Supplementary file 2 — Figure S1 [file 41396_2020_588_MOESM2_ESM.pdf]

Figure S2

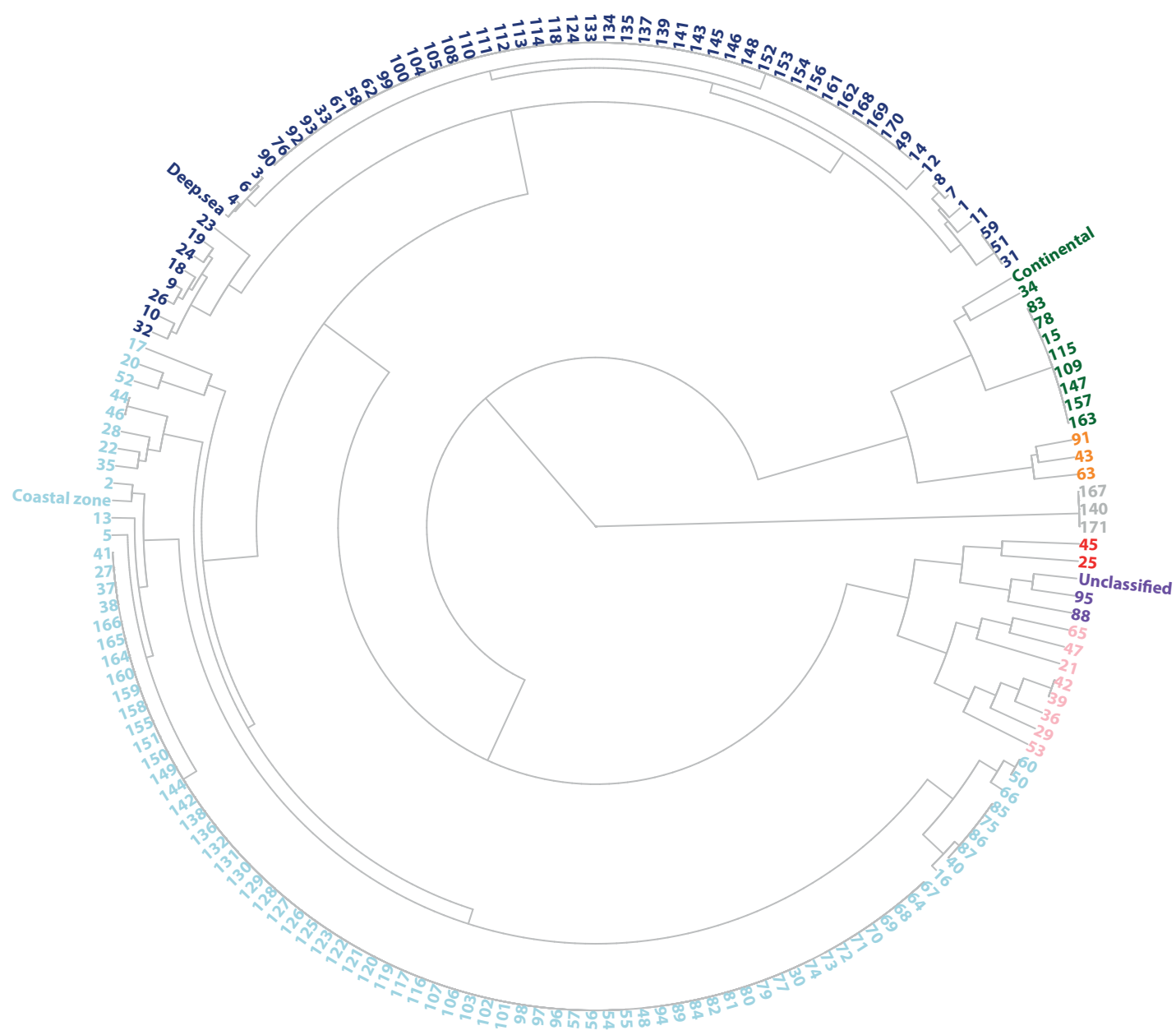

Supplement: Supplementary file 3 — Figure S2 [file 41396_2020_588_MOESM3_ESM.pdf]

Figure S3

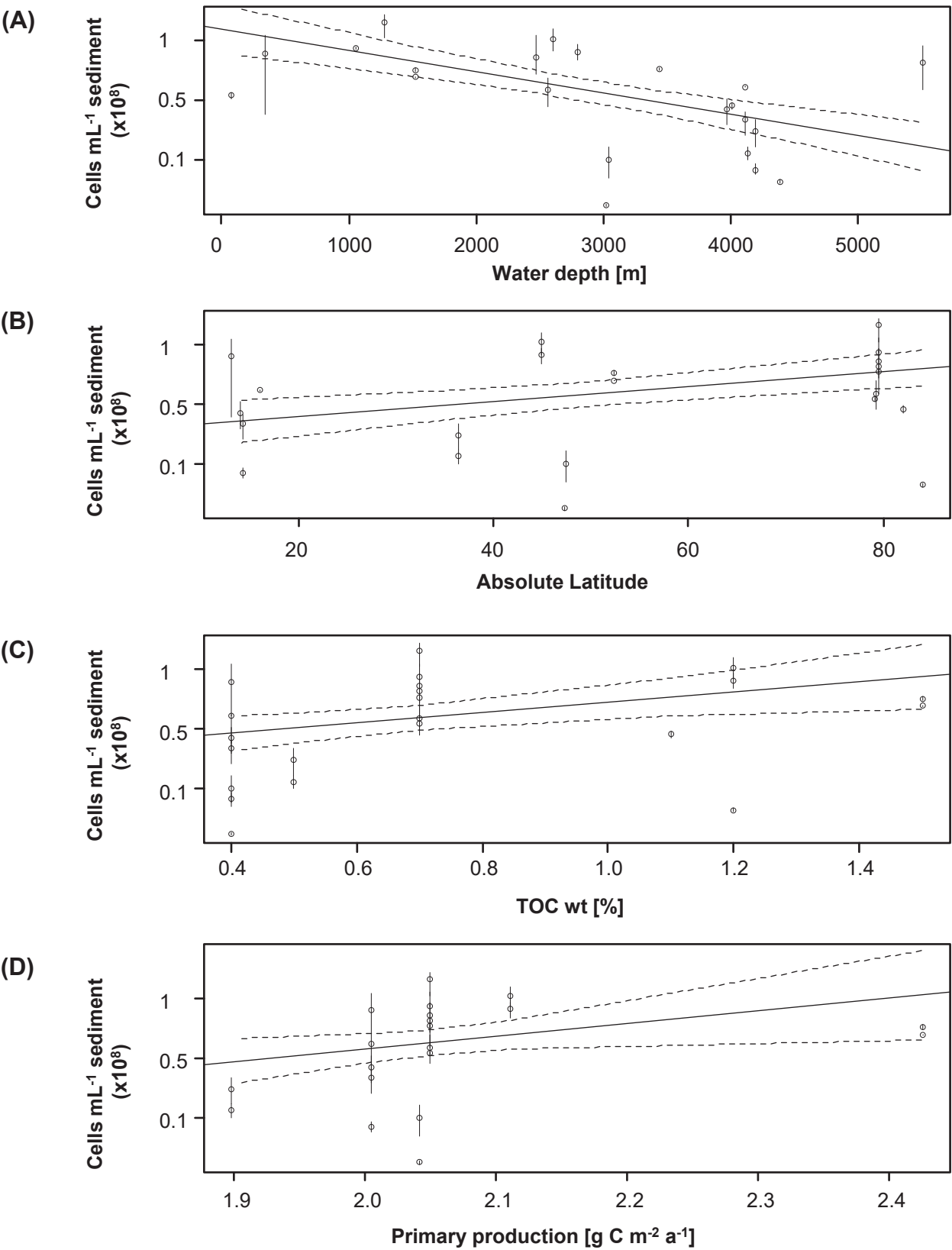

Supplement: Supplementary file 4 — Figure S3 [file 41396_2020_588_MOESM4_ESM.pdf]

Figure S4

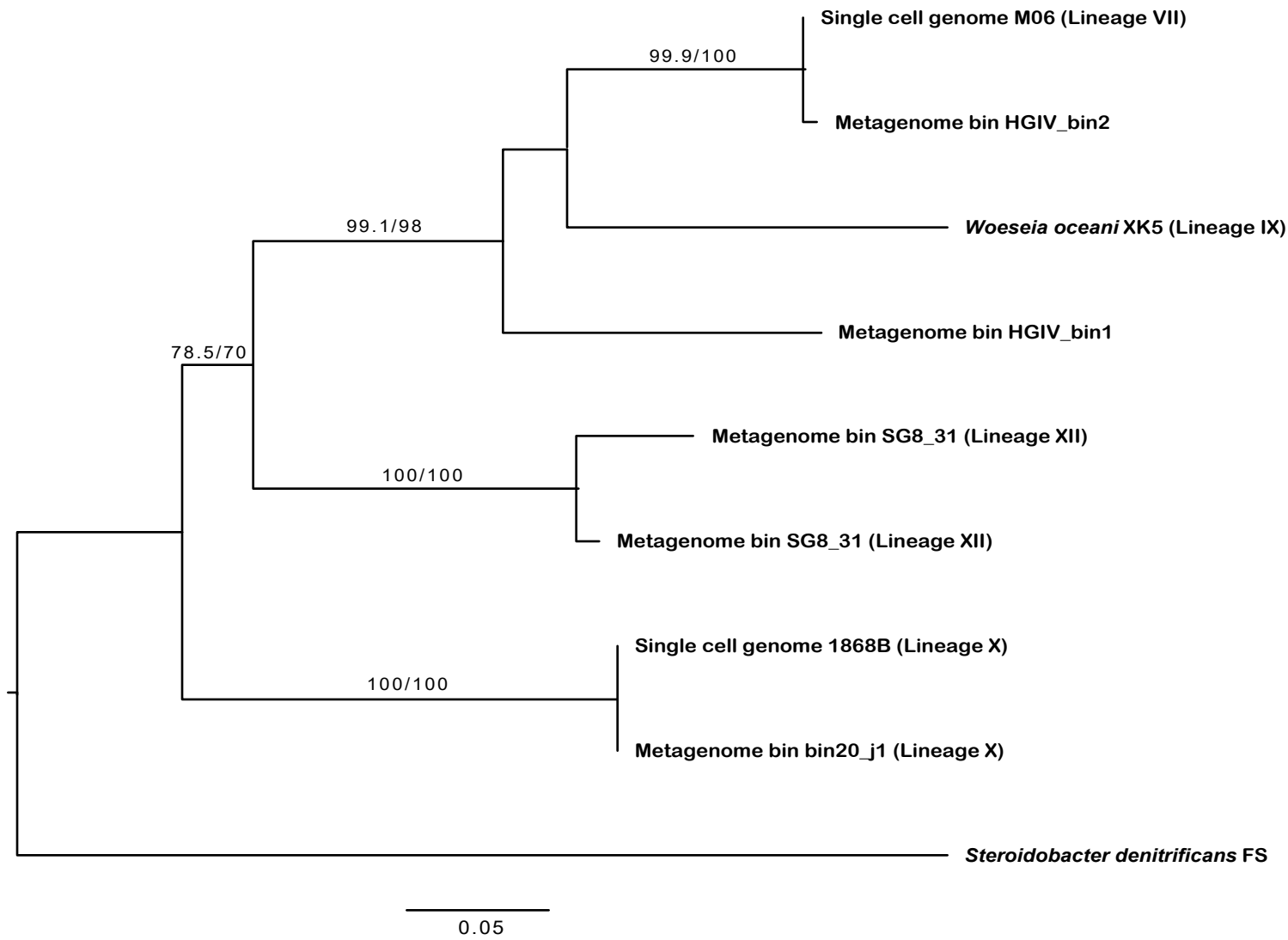

Supplement: Supplementary file 5 — Figure S4 [file 41396_2020_588_MOESM5_ESM.pdf]
