# Supplementary material for: Diversity and metabolism of Woeseiales bacteria, global members of marine sediment communities
Source: ISME J. 2020 Jan 27;14(4):1042–56. doi: 10.1038/s41396-020-0588-4 (PMC7082342; doi:10.1038/s41396-020-0588-4)
Supplement: Supplementary file 26 — Supplementary file 6 [file 41396_2020_588_MOESM26_ESM.pdf]

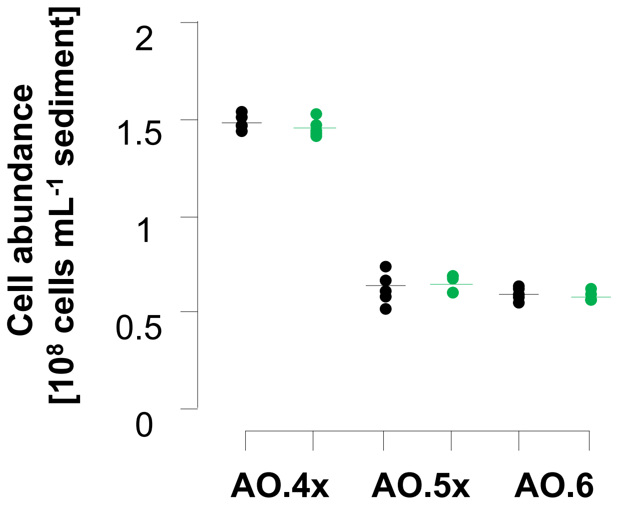

Supplementary file 6. Abundance of cells detected by a mix of oligonucleotides including the CARD-FISH probes JTB819, JTB897 and competitor oligonucleotide cJTB897 (Table S3) in sonicated (black dots) and non-sonicated (green dots) sediment samples originating from three stations of the LTER HAUSGARTEN (see Table S1 for the characteristics of the sampling sites). The horizontal black lines indicate mean values of 5 replicates.
