# Supplementary material for: Diversity and metabolism of Woeseiales bacteria, global members of marine sediment communities
Source: ISME J. 2020 Jan 27;14(4):1042–56. doi: 10.1038/s41396-020-0588-4 (PMC7082342; doi:10.1038/s41396-020-0588-4)
Supplement: Supplementary file 28 — Supplementary file 8 [file 41396_2020_588_MOESM28_ESM.pdf]

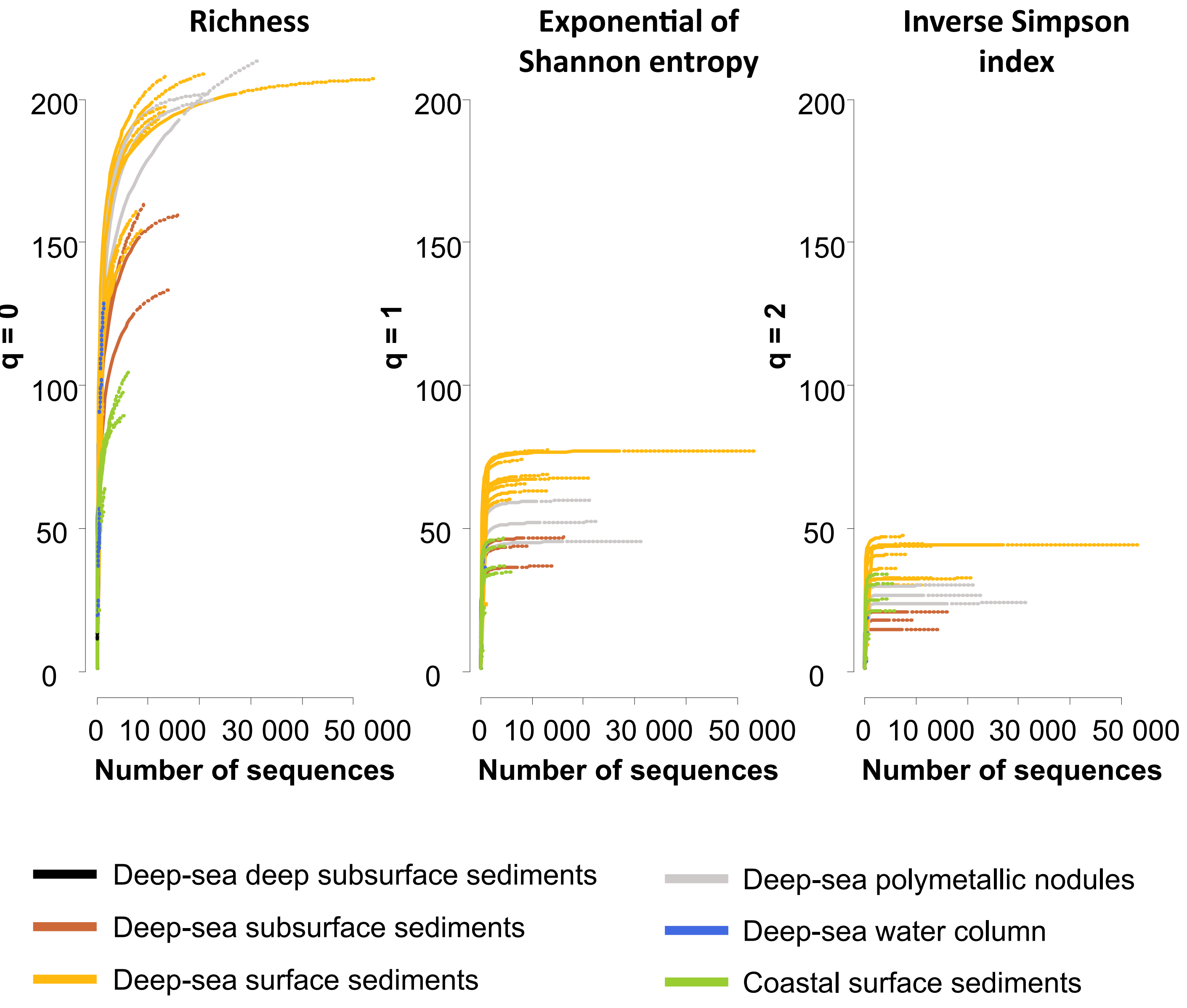

Supplementary file 8. Rarefaction curves illustrating the diversity of oligotypes generated from 16S rRNA gene sequence tags assigned to the JTB255 clade. Each of the three plots represent a distinct measure of the diversity of oligotypes. Oligotype profiles are shown in Figure 4. The sequence tags were obtained from a variety of marine sediment and water samples as indicated directly on the figure by the color key. Analysed samples are specified in Figure 4 and sample descriptions are provided in Table S1. Rarefaction curves were generated with the iNEXT package (Hsieh *et al.* 2016).
